# A tri-target in silico analysis of Testolift: a nutraceutical formulation targeting aromatase, myostatin, and prolyl hydroxylase-2 in testosterone regulation and muscle performance

**DOI:** 10.1038/s41598-026-57541-y

**Published:** 2026-06-11

**Authors:** Augustine Amalraj, V. Anantha Narayanan, Kaniyath Ramachandran Reshna, Karthik Varma, Preetha Balakrishnan, Sreerag Gopi

**Affiliations:** 1 NIMP Innovation Hub, Padmalife Nutrition Private Limited, Koratty, Thrissur, Kerala 680 309 India; 2 Global Innovation Centre, Molecules Biolabs Private Limited, Koratty, Thrissur, Kerala 680 309 India

**Keywords:** Testolift, Nutraceutical formulation, Testosterone metabolism, Molecular docking, Molecular dynamics simulation, Biochemistry, Computational biology and bioinformatics, Drug discovery, Structural biology

## Abstract

Declining testosterone levels and associated impairments in muscle function, endurance, and metabolic health represent a growing global concern, while limitations associated with long-term testosterone replacement therapy have intensified interest in safer, mechanism-based nutraceutical approaches. In the present study, a systems-oriented multi-target computational framework integrating physicochemical profiling, ADMET prediction, molecular docking, and molecular dynamics (MD) simulations was employed to investigate the mechanistic basis of the Testolift formulation composed of protodioscin, diosgenin, 5,7-dimethoxyflavone (DMF), and 5,7,4′-trimethoxyflavone (TMF). The phytochemicals were evaluated against three mechanistically interconnected targets namely aromatase (CYP19A1), myostatin, and prolyl hydroxylase-2 (PHD2), which are associated with testosterone regulation, muscle physiology, and oxygen-dependent metabolic adaptation. Protodioscin demonstrated extensive polar interactions and persistent hydrogen bonding within the CYP19A1 binding region, whereas diosgenin exhibited favorable lipophilicity and interaction characteristics consistent with steroidogenic signaling. DMF and TMF displayed the most favorable drug-like physicochemical profiles (QED = 0.74), high predicted intestinal absorption, and stable interactions with myostatin and PHD2. Molecular dynamics simulations performed over 150 ns confirmed stable ligand–protein complexes without major conformational destabilization. ADMET analysis further indicated complementary pharmacokinetic behavior together with an overall favorable predicted safety profile. Collectively, these findings support a coordinated multi-target nutraceutical framework in which structurally distinct phytochemicals modulate complementary peripheral pathways associated with testosterone balance, muscle anabolic regulation, and endurance-related metabolic adaptation, thereby supporting further experimental validation of the Testolift formulation.

## Introduction

Testosterone is a central androgenic hormone regulating male reproductive physiology, skeletal muscle anabolism, bone remodeling, erythropoiesis, metabolic homeostasis, and overall physical vitality. In recent decades, a progressive global decline in testosterone has been documented across multiple populations, affecting not only older men but increasingly younger age groups as well. Large epidemiological studies from Europe, North America, and Asia demonstrate a 10–40% prevalence of testosterone deficiency (TD), with clear secular downward trends even after adjusting for confounding factors such as age, obesity, comorbidities, and medication use^1–4^. Region-specific analyses further indicate a disproportionate rise in TD in South Asian and Middle Eastern countries, where rapid urbanization, environmental pollution, chronic stress, and dietary transitions contribute to androgen suppression^4^. Even mild reductions in testosterone within the lower quartile of the reference range have clinically meaningful associations with sexual dysfunction, impaired muscle mass and endurance, abdominal adiposity, reduced metabolic flexibility, depressed mood, and increased cardiometabolic risk^5^.

Although testosterone replacement therapy (TRT) remains a widely used clinical approach, it presents significant drawbacks that limit its applicability as a long-term strategy. Several large observational and interventional studies have described increased risks of erythrocytosis, cardiovascular events, venous thromboembolism, infertility due to suppression of the hypothalamic–pituitary–gonadal (HPG) axis, and concerns regarding prostate health^6–8^. These limitations have intensified interest in non-hormonal, mechanism-based nutraceutical interventions that enhance endogenous testosterone production, preserve androgen availability, and support muscle performance without disrupting physiological feedback regulation.

Testosterone regulation is influenced by multiple interconnected pathways including central HPG signaling, aromatase-mediated testosterone conversion, 5α-reductase metabolism, sex hormone-binding globulin (SHBG) dynamics, redox and inflammatory status, mitochondrial energetics, and oxygen-sensing mechanisms^9–12^. While many of these pathways play important biological roles, not all are ideal targets for nutraceutical modulation. Manipulating the HPG axis can lead to unpredictable endocrine responses; inhibiting 5α-reductase alters the testosterone-to-dihydrotestosterone ratio with possible androgenic side effects; and SHBG modulation is tightly regulated and difficult to influence predictably using natural compounds. Therefore, peripheral mechanisms that regulate testosterone availability, skeletal muscle responsiveness, and metabolic/oxygenation efficiency present a more rational and safer basis for nutraceutical development.

Based on this rationale, the present work focuses on three mechanistically complementary biological targets such as CYP19A1 (aromatase), Myostatin, and Prolyl Hydroxylase-2 (PHD2), each of which addresses a distinct component of testosterone biology, muscle performance, and endurance capacity. Aromatase (CYP19A1) converts testosterone to estradiol, and increased aromatase activity has been associated with reduced circulating testosterone levels under conditions such as obesity, inflammation, and environmental stress^13,14^. Modulation of aromatase-associated pathways therefore represents a biologically relevant approach for supporting endogenous testosterone balance without directly interfering with upstream endocrine regulation. In support of this rationale, de Ronde and de Jong^15^ reported that aromatase inhibition in men may contribute to preservation of testosterone availability and altered androgen-estrogen balance through reduction of testosterone-to-estradiol conversion.

Myostatin, a member of the TGF-β superfamily, is a potent inhibitor of skeletal muscle hypertrophy, protein synthesis, and mitochondrial biogenesis. Elevated myostatin levels, particularly in aging and metabolic syndrome, impair anabolic signaling and reduce functional responses to testosterone^13,16^. Modulation of myostatin-associated pathways has been linked with improvements in muscle mass, strength, and exercise-related physiological adaptation. Supporting this concept, Sharp et al.^17^ demonstrated that myostatin inhibition was associated with increases in lean body mass, strength, and muscular performance in resistance-trained individuals.

Prolyl Hydroxylase-2 (PHD2) is a key oxygen-sensing enzyme that regulates the degradation of hypoxia-inducible factor-1α (HIF-1α). Excess PHD2 activity reduces HIF-1α stability and suppresses pathways involved in angiogenesis, nitric oxide synthesis, mitochondrial function, and erythropoiesis. Modulation of PHD2-associated signaling may support cellular oxygen utilization, mitochondrial bioenergetics, and endurance-related metabolic adaptation. Because Leydig-cell steroidogenesis is closely associated with mitochondrial function and oxygen availability, PHD2–HIF pathway modulation may also contribute indirectly to testosterone biosynthetic processes^18–22^.

Testolift, a testosterone-supporting nutraceutical formulation, was developed using saponin-rich fenugreek (*Trigonella foenum-graecum*) extract and methoxyflavone-rich black ginger (*Kaempferia parviflora*) extract, combined with zinc methionine, with the intent of supporting testosterone balance, muscle maintenance, and vascular adaptation. The tri-target approach involving CYP19A1 for hormonal balance, Myostatin for muscle anabolic regulation, and PHD2 for oxygen-dependent endurance physiology offers a comprehensive strategy to address testosterone decline and performance-associated physiological deficits. These targets were selected to align with the pharmacological properties of the chosen nutraceutical ingredients: fenugreek saponins, particularly protodioscin and diosgenin, which support testosterone synthesis and exhibit aromatase-modulating potential^13,23^; and black ginger polymethoxyflavones, including 5,7-dimethoxyflavone and 5,7,4′-trimethoxyflavone, which have been associated with nitric oxide production, mitochondrial bioenergetics, myostatin suppression, and endurance-related physiological effects^13,14,16^. Zinc methionine, a bioavailable source of zinc, further supports the formulation through its role in steroidogenic enzyme function and antioxidant protection of testicular tissue^24,25^. These bioactive constituents were co-formulated using the InSitu360 molecular complexation technology, which improves bioactive dispersibility, molecular stability, and coordinated delivery of structurally distinct phytochemicals. This molecular-level formulation strategy supports efficient incorporation of fenugreek saponins, polymethoxyflavones, and zinc within a mechanistically integrated nutraceutical system.

The phytochemicals selected for the present computational investigation namely protodioscin, diosgenin, 5,7-dimethoxyflavone (DMF), and 5,7,4′-trimethoxyflavone (TMF) represent major bioactive constituents associated with the biological activities of fenugreek and black ginger extracts. These compounds were selected based on their reported involvement in steroidogenic modulation, mitochondrial bioenergetics, nitric oxide signaling, and muscle-performance–related physiological pathways. Accordingly, the present study was designed to evaluate the mechanistic relevance of biologically representative constituents present within the Testolift formulation rather than isolated theoretical compounds without formulation context.

To substantiate these mechanistic hypotheses, the present study employs an in silico multi-target docking and molecular dynamics strategy to evaluate the binding interactions and dynamic behavior of protodioscin, diosgenin, 5,7-dimethoxyflavone, and 5,7,4′-trimethoxyflavone against selected targets including CYP19A1, Myostatin, and PHD2. This integrative computational approach was designed to investigate whether structurally diverse phytochemicals within the Testolift formulation exhibit coordinated interaction profiles across mechanistically interconnected pathways relevant to testosterone regulation, muscle physiology, and oxygen-dependent metabolic adaptation.

Despite growing interest in nutraceutical interventions for testosterone support and muscle performance, most previous studies have primarily focused on isolated phytochemicals, single-target mechanisms, or experimental observations without integrating multi-target molecular interaction analysis, pharmacokinetic characterization, and dynamic stability assessment within a unified computational framework^26–28^. Recent reviews and clinical investigations have demonstrated the beneficial effects of fenugreek-derived saponins and black ginger polymethoxyflavones on testosterone regulation, muscle performance, endurance, and metabolic adaptation^29–31^; however, the coordinated molecular interaction behavior of these phytochemicals across interconnected targets such as CYP19A1, Myostatin, and PHD2 remains insufficiently characterized.

To address these gaps, the present study integrates physicochemical profiling, ADMET prediction, molecular docking, and molecular dynamics simulations to systematically investigate the coordinated multi-target interaction potential of major Testolift phytochemicals against CYP19A1, Myostatin, and PHD2. Within this framework, ADMET analysis was employed to evaluate the physicochemical and pharmacokinetic suitability of the investigated phytochemicals, molecular docking was used to characterize target-specific binding interactions, and molecular dynamics simulations were performed to assess the stability and dynamic behavior of the resulting protein–ligand complexes under physiologically relevant conditions. The methodological contribution of this work lies in the application of a systems-oriented computational framework designed to evaluate complementary pathway modulation by structurally distinct nutraceutical bioactives rather than focusing exclusively on single-target affinity optimization.

**Fig. 1:**
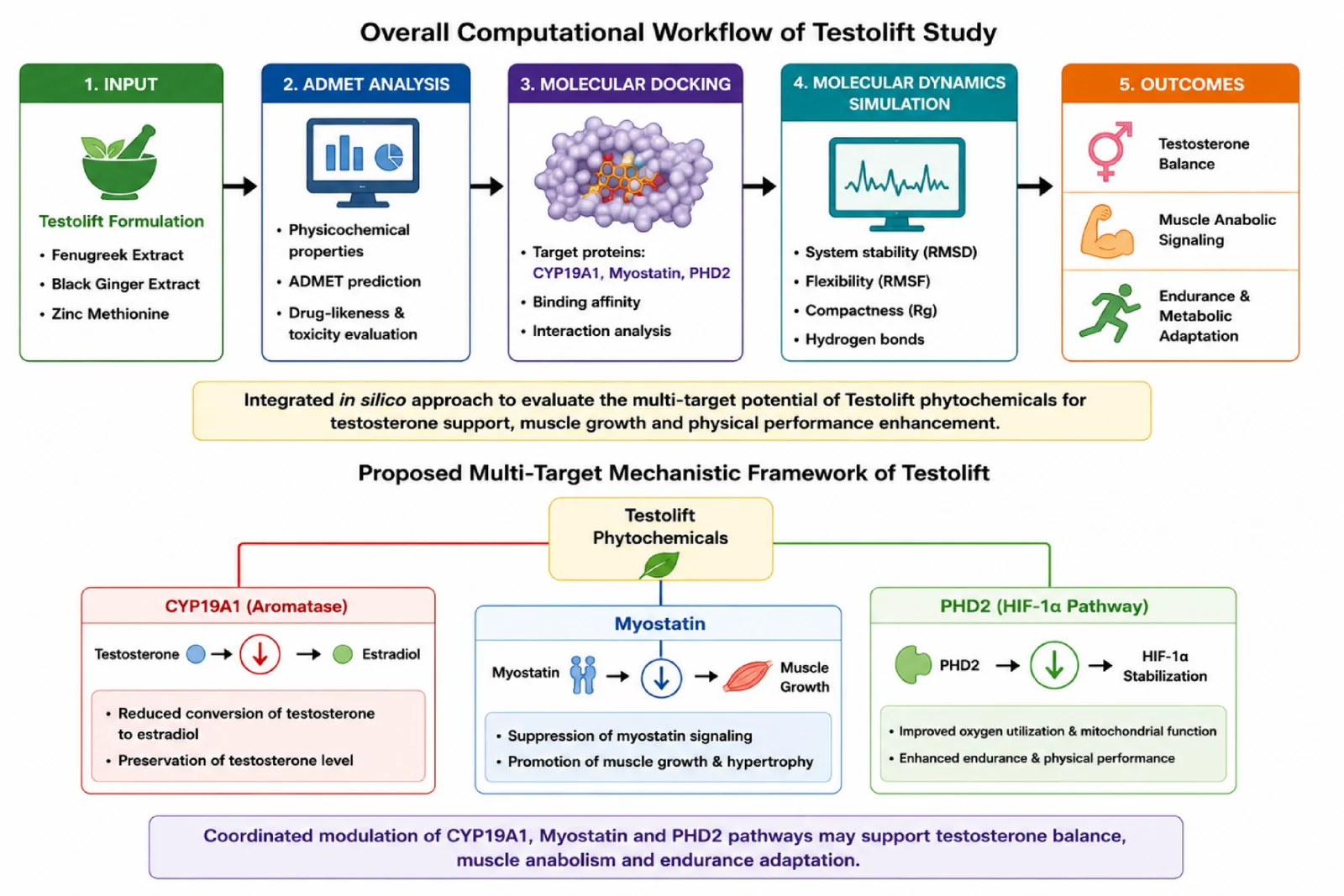
Overall computational workflow and proposed multi-target mechanistic framework of the Testolift formulation. The study integrates ADMET prediction, molecular docking, and molecular dynamics simulations to evaluate the coordinated interaction potential of Testolift phytochemicals against CYP19A1, Myostatin, and PHD2 pathways associated with testosterone regulation, muscle anabolic signaling, and endurance-related metabolic adaptation.

Unlike conventional single-target pharmacological strategies aimed at maximizing affinity toward an isolated receptor, the Testolift formulation was designed as a systems-oriented nutraceutical approach intended to modulate multiple peripheral pathways associated with testosterone regulation, muscle anabolic signaling, and endurance-related metabolic adaptation. Accordingly, the objective of the present investigation was not to identify a single high-affinity ligand, but rather to evaluate the potential for coordinated multi-target engagement through structurally diverse phytochemicals. Such an approach may be particularly relevant in nutraceutical systems, where moderate yet coordinated interactions across interconnected biological targets can collectively contribute to physiological effects associated with testosterone preservation, muscle performance, and metabolic adaptation. The overall computational workflow together with the proposed multi-target mechanistic framework of the Testolift formulation is illustrated in Fig. 1.

## Materials and methods

### Testolift formulation and selection of representative bioactive compounds

Testolift is a nutraceutical formulation composed of fenugreek (*Trigonella foenum-graecum*) extract enriched in steroidal saponins, black ginger (*Kaempferia parviflora*) extract enriched in polymethoxyflavones, and zinc methionine. The formulation was developed using the proprietary InSitu360 molecular complexation technology to improve dispersibility, molecular stabilization, and coordinated delivery of structurally distinct phytochemicals. The compounds selected for the present in silico investigation namely protodioscin, diosgenin, 5,7-dimethoxyflavone (DMF), and 5,7,4′-trimethoxyflavone (TMF) were chosen as representative bioactive constituents based on their abundance within the botanical ingredients and their reported roles in testosterone regulation, muscle physiology, mitochondrial function, and endurance-related metabolic pathways.

### In silico ADMET and toxicity prediction

The pharmacokinetic and toxicity profiles of the selected phytochemicals such as diosgenin (PubChem CID: 99474), protodioscin (PubChem CID: 441891), 5,7-dimethoxyflavone (DMF; PubChem CID: 88881), and 5,7,4′-trimethoxyflavone (TMF; PubChem CID: 79730) were evaluated using the ADMET-AI web server (https://admet.ai/)^32^. Canonical SMILES representations of all compounds were retrieved from the PubChem database to ensure standardized molecular input^33^. ADMET-AI employs machine-learning–based predictive models trained on large experimental datasets to estimate absorption, distribution, metabolism, excretion, and toxicity parameters.

Predicted absorption-related descriptors included human intestinal absorption, aqueous solubility, lipophilicity (log P), and P-glycoprotein interaction, providing insight into the oral uptake and intestinal transport characteristics of the selected compounds. Distribution-related parameters comprised blood–brain barrier permeability, plasma protein binding, and volume of distribution, reflecting systemic exposure and tissue dissemination potential. Metabolic liabilities were evaluated through predictions of cytochrome P450 enzyme inhibition and substrate likelihood, including the major isoforms CYP1A2, CYP2C9, CYP2C19, CYP2D6, and CYP3A4. Excretion-related properties such as clearance and biological half-life were also estimated. Toxicological endpoints encompassed hepatotoxicity, carcinogenicity, AMES mutagenicity, hERG potassium channel inhibition, acute toxicity, and drug-induced liver injury. Collectively, these in silico predictions enabled a comprehensive comparative assessment of drug-likeness, pharmacokinetic feasibility, and safety profiles of the selected phytochemicals prior to molecular docking and molecular dynamics simulations.

### Ligand preparation

The ligand dataset used in the present study consisted of four major bioactive phytochemicals associated with the Testolift formulation namely diosgenin, protodioscin, DMF, and TMF. Two-dimensional chemical structures of diosgenin, protodioscin, DMF, and TMF were retrieved from the NCBI PubChem database in Structure Data File (SDF) format^33^. The ligands were converted into three-dimensional structures and subsequently transformed into Protein Data Bank (PDB) format using Open Babel^34^. Further preparation involved conversion to PDBQT format using AutoDock Tools (ADT) version 1.5.7 within the MGLTools suite. Energy minimization and geometric optimization were performed using default parameters implemented in Open Babel via the PyRx virtual screening platform to relieve steric clashes and obtain energetically favorable conformations. Gasteiger charges were assigned, polar hydrogens were added, and rotatable bonds were defined to allow conformational flexibility during docking simulations^35,36^. The prepared ligand structures were subsequently utilized for physicochemical profiling, ADMET prediction, molecular docking, and molecular dynamics simulation analyses.

### Protein preparation

The protein dataset used in the present investigation consisted of aromatase (CYP19A1; PDB ID: 5JKV), myostatin (GDF-8; PDB ID: 5F3B), and prolyl hydroxylase domain protein-2 (PHD2; PDB ID: 2G19), which were retrieved from the RCSB Protein Data Bank and prepared for molecular docking and molecular dynamics simulations using AutoDock Tools. CYP19A1 is a heme-dependent cytochrome P450 monooxygenase responsible for the aromatization of androgens to estrogens. Its active site comprises a deeply buried hydrophobic cavity surrounding a prosthetic heme group, with catalytically important residues including the heme-ligating cysteine (Cys437) and residues involved in substrate orientation and electron transfer such as Arg375. Proper representation of this active site is critical for accurate docking of steroidal and polyphenolic ligands^37^. Myostatin (GDF-8), a member of the transforming growth factor-β (TGF-β) superfamily, regulates skeletal muscle growth through receptor-mediated signaling. The biologically relevant binding regions are located within the mature growth factor domain, which is structurally governed by the cystine-knot fold and regulated by latency-associated prodomain elements. Structural features such as the β6′ and β7′ strands and adjacent surface loops are essential for ligand and receptor interactions^38^. PHD2 is a Fe (II)/2-oxoglutarate–dependent dioxygenase that hydroxylates HIF-α, thereby regulating oxygen sensing and cellular adaptation to hypoxia. The active site of PHD2 is embedded within a double-stranded β-helix (DSBH) fold and contains a conserved iron-coordinating triad composed of His313, Asp315, and His374. This catalytic pocket is predominantly hydrophobic and incorporates a flexible β2–β3 loop that undergoes conformational closure upon substrate binding, a structural feature that is essential for proper substrate positioning and enzymatic activity^39^. For all target proteins, crystallographic water molecules were removed to avoid non-specific interactions during docking simulations. Polar hydrogens were added, Kollman partial charges were assigned, and missing atoms or residues were repaired where necessary to ensure structural completeness. The prepared protein structures were subsequently saved in PDBQT format for use in molecular docking analyses^40^. The prepared protein structures were subsequently utilized for molecular docking, interaction profiling, and molecular dynamics simulation studies.

### Molecular docking

Molecular docking was performed to investigate the binding affinity and interaction patterns of the phytochemicals with CYP19A1, Myostatin, and PHD2. Docking simulations were conducted using the PyRx virtual screening platform employing the AutoDock Vina docking engine for binding affinity prediction and docking pose analysis. All investigated phytochemicals were independently docked against each selected target protein to enable comparative evaluation of binding affinity, interaction architecture, and target-specific interaction behavior across the CYP19A1, Myostatin, and PHD2 systems. Grid boxes were centered on the respective active sites of each protein and sized to fully encompass all key catalytic and regulatory residues, ensuring adequate sampling of the binding pocket^37–39^. For CYP19A1, the grid center was set at X = 60, Y = 74, Z = 58, with grid box dimensions of X = 84.2126, Y = 54.483, and Z = 47.8934. For Myostatin, the grid center was defined at X = 64, Y = 76, Z = 58, with grid box dimensions of X = 82.97, Y = 54.25, and Z = 47.77. For PHD2, the grid center was positioned at X = 62, Y = 58, Z = 48, and the grid box dimensions were X = –0.0747, Y = 41.8193, and Z = 22.2842. A uniform grid spacing of 1.00 Å was applied across all docking runs. Docking parameters were kept consistent for all ligands and targets to enable direct comparison of binding energies and interaction profiles. The binding affinity predictions generated during molecular docking were based on the AutoDock Vina scoring function, which estimates the binding free energy (ΔG) of protein–ligand complexes using empirical steric, hydrophobic, hydrogen-bonding, and conformational interaction terms according to Eq. (1):

1 $$\:\varDelta\:G={\sum\:}_{i<j}[w1Gauss1\left(rij\right)+w2Gauss2\left(rij\right)+w3Repulsion\left(rij\right)+w4Hydrophobic(rij)+w5HydrogenBond(rij\left)\right]+w6Nrot$$

where ΔG represents the predicted binding free energy; rij denotes the interatomic distance between atoms i and j; Gauss1 and Gauss2 correspond to steric attractive interaction terms; Repulsion represents steric clash penalties; Hydrophobic corresponds to hydrophobic interaction contributions; Hydrogen Bond represents hydrogen-bonding interactions; Nrot denotes the torsional penalty associated with ligand flexibility; and w1–w6 represent empirically optimized weighting coefficients implemented in AutoDock Vina. This scoring framework enables estimation of ligand binding stability and interaction favorability within the selected protein binding regions^41^. The best-ranked binding poses were selected based on the lowest predicted binding free energy together with favorable interaction geometry and were subsequently used for molecular interaction analysis and molecular dynamics simulations.

### Molecular dynamics simulation

The top-ranked protein–ligand complexes selected from molecular docking analyses based on predicted binding affinity and interaction geometry were subsequently subjected to molecular dynamics (MD) simulations to evaluate structural stability and dynamic interaction behavior under physiologically relevant simulation conditions. Ligand topology files were generated using the SWISS-PARAM server to ensure compatibility with the CHARMM force field. Protein topologies were constructed using the CHARMM27 force field within GROMACS version 2024.5. The TIP3P water model was used to solvate the systems. Each complex was placed in a triclinic simulation box with a minimum distance of 1.0 nm between the protein surface and box edges. Systems were solvated using the SPC216 water model and electrically neutralized by addition of appropriate Na⁺ and Cl⁻ counter ions using the gmx genion module to achieve an ionic strength of 0.1 M, approximating physiological conditions. The number of ions varied among the systems depending on the net charge and protonation state of each protein–ligand complex. Accordingly, a total of 43 ions were added for the PHD2 system, 58 ions for the myostatin system, and 71 ions for the CYP19A1 system prior to simulation.

Energy minimization was performed using the steepest descent algorithm until the maximum force dropped below 1000 kJ·mol^− 1^·nm^− 1^. Equilibration was carried out in two phases: a 100 ps NVT ensemble to stabilize temperature, followed by a 100 ps NPT ensemble to equilibrate pressure and density, with positional restraints applied to heavy atoms. Production MD simulations were then conducted for 150 ns under periodic boundary conditions using the leapfrog integrator. Temperature (300 K) and pressure (1 bar) were maintained using the V-rescale thermostat and Parrinello–Rahman barostat, respectively. Long-range electrostatic interactions were calculated using the Particle Mesh Ewald (PME) method, and all covalent bonds were constrained using the LINCS algorithm. Post-simulation trajectory analyses were performed using standard GROMACS tools to calculate root mean square deviation (RMSD), root mean square fluctuation (RMSF), radius of gyration (Rg), and hydrogen bond dynamics. These analyses provided quantitative insights into global stability, residue-level flexibility, protein compactness, and ligand–protein interaction persistence across all simulated complexes^42,43^.

## Results and discussion

### Physicochemical evaluation of key phytochemicals in the Testolift formulation

The physicochemical analysis of the principal bioactives in the Testolift formulation revealed substantial structural diversity among the investigated phytochemicals (Table 1). Protodioscin exhibited the highest molecular weight (1049.21 g/mol), together with high polarity, reflected by a negative cLogP value (–1.98) and a large topological polar surface area (TPSA) of 346.06 Å². The compound also demonstrated extensive hydrogen-bonding capacity with 22 hydrogen-bond acceptors (HBA) and 13 hydrogen-bond donors (HBD), consistent with the structural complexity of glycosylated steroidal saponins. In contrast, diosgenin displayed a comparatively lower molecular weight (414.63 g/mol), high lipophilicity (cLogP 5.71), and low TPSA (38.69 Å²), supporting its compact steroidal architecture and membrane-associated interaction potential. The polymethoxyflavones DMF and TMF exhibited lower molecular weights, balanced lipophilicity (cLogP ~ 3.5), and moderate TPSA values, together with the highest QED scores (0.74) among the investigated compounds, indicating physicochemical properties favorable for intracellular target engagement.

Collectively, these physicochemical characteristics demonstrate clear distinctions among the investigated phytochemical classes and support their complementary functional roles within the Testolift formulation. The high polarity and extensive hydrogen-bonding potential of protodioscin are consistent with interaction behavior associated with extracellular or membrane-associated targets such as CYP19A1^44,45^. In contrast, the lipophilic steroidal framework of diosgenin supports membrane-associated steroidogenic interactions relevant to testosterone biosynthesis^46–49^. The polymethoxyflavones DMF and TMF exhibited physicochemical profiles compatible with intracellular accessibility and interaction with signaling-associated targets involved in muscle physiology, mitochondrial bioenergetics, and oxygen-dependent metabolic pathways^13,16,28^. Taken together, the ADMET-derived physicochemical profiles support a coordinated multi-component framework in which structurally distinct phytochemicals contribute complementary properties relevant to testosterone regulation, muscle anabolic signaling, and endurance-associated metabolic adaptation within the Testolift formulation.

**Table 1:** In silico ADMET-AI predicted physicochemical properties of the key phytochemicals present in the Testolift formulation, including protodioscin and diosgenin from fenugreek, and 5,7-dimethoxyflavone (DMF) and 5,7,4′-trimethoxyflavone (TMF) from black ginger.

Property	Protodioscin	Diosgenin	DMF	TMF
Molecular weight (g/mol)	1049.21	414.63	282.29	312.32
cLogP	-1.98	5.71	3.48	3.49
Hydrogen bond acceptors (HBA)	22	3	4	5
Hydrogen bond donors (HBD)	13	1	0	0
Lipinski rule of 5	1	3	4	4
Quantitative estimate of drug likeness (QED)	0.08	0.52	0.74	0.74
Stereo centers	31	11	0	0
Topological polar surface area (Å²)	346.06	38.69	48.67	57.9

### In silico absorption profiling of Testolift bioactives and their pharmacokinetic implications

The ADMET-AI absorption evaluation revealed distinct differences in intestinal uptake, membrane permeability, and bioavailability among the Testolift bioactive compounds (Table 2). Protodioscin exhibited the lowest predicted human intestinal absorption (HIA = 0.21), oral bioavailability (0.26), and PAMPA permeability (0.22), together with unfavorable cell permeability and hydration energy values, consistent with the highly polar and glycosylated nature of steroidal saponins. In the context of the Testolift formulation, such physicochemical limitations were among the considerations underlying the incorporation of the InSitu360 bioefficacy enhancement platform, which was designed to improve molecular dispersion, gastrointestinal compatibility, and overall bioefficacy of structurally complex phytochemical constituents. Future formulation optimization strategies including molecular complexation, phospholipid-based delivery systems, nano-dispersion approaches, and lipid-associated nutraceutical platforms may further improve the gastrointestinal performance and systemic availability of protodioscin-containing formulations. In contrast, diosgenin demonstrated high intestinal absorption (HIA = 1.0), favorable oral bioavailability (0.87), and strong membrane permeability characteristics, reflecting its lipophilic steroidal structure. Similarly, the polymethoxyflavones DMF and TMF exhibited high intestinal absorption, favorable oral bioavailability, and excellent PAMPA permeability, together with cell permeability values indicative of efficient membrane traversal and intracellular accessibility. Differences in aqueous solubility and P-glycoprotein (P-gp) interaction profiles further reflected the structural diversity of the investigated phytochemicals. Protodioscin displayed comparatively greater aqueous solubility and lower predicted P-gp inhibition, whereas diosgenin, DMF, and TMF exhibited lower aqueous solubility and higher predicted interaction with efflux transporters, consistent with their increased lipophilicity and membrane-associated behavior.

Collectively, the absorption-related properties of the Testolift phytochemicals support complementary pharmacokinetic characteristics within the formulation. The limited permeability and extracellular interaction tendency of protodioscin are consistent with surface-associated or peripheral target engagement^26,50–52^, whereas diosgenin exhibited properties compatible with membrane-associated steroidogenic interactions and intracellular accessibility^50^. The polymethoxyflavones DMF and TMF demonstrated absorption profiles favorable for intracellular target engagement, aligning with previous observations regarding the efficient membrane permeability of methoxylated flavonoids^53,54^. Together, these findings support a coordinated multi-component framework in which structurally distinct phytochemicals exhibit complementary absorption and permeability characteristics relevant to testosterone regulation, muscle physiology, and oxygen-dependent metabolic adaptation^55–58^.

**Table 2:** ADMET AI absorption analysis.

Property	Protodioscin	Diosgenin	DMF	TMF
Human intestinal absorption (HIA)	0.21	1	1	1
Oral bioavailability	0.26	0.87	0.85	0.87
Aqueous solubility	-3.76	-6.36	-5.54	-5.41
Lipophilicity	1.96	4.17	3.3	3.38
Hydration free energy (kcal/mol)	-11.37	-3.82	-7.9	-8.3
Cell effective permeability	-6.62	-5.15	-4.4	-4.45
PAMPA permeability	0.22	0.97	0.99	0.98
P-glycoprotein inhibition	0.36	0.85	0.85	0.87

### Metabolic liabilities and CYP450 interactions supporting the multi-target mechanism of Testolift

The ADMET-AI metabolism analysis revealed distinct CYP450 interaction profiles among the Testolift bioactives, reflecting differences in their structural classes and predicted metabolic behavior (Table 3). Protodioscin exhibited minimal predicted inhibition across major CYP isoforms, together with low substrate probabilities for CYP2C9 and CYP2D6, indicating limited interaction with hepatic metabolic pathways. In contrast, diosgenin demonstrated moderate CYP interaction probabilities, particularly involving CYP3A4, consistent with the oxidative metabolism commonly associated with steroidal compounds. The polymethoxyflavones DMF and TMF exhibited comparatively higher inhibition and substrate probabilities across multiple CYP isoforms, especially CYP1A2, CYP2C19, and CYP3A4, suggesting greater interaction with hepatic metabolic systems.

These metabolic differences align closely with the structural characteristics of the investigated phytochemicals. The low CYP interaction profile of protodioscin is consistent with the high polarity and glycosylated architecture of steroidal saponins, which generally exhibit limited CYP binding affinity and reduced hepatic metabolism^59–61^. In contrast, the steroidal framework and lipophilic nature of diosgenin support moderate CYP-mediated oxidative metabolism, particularly through CYP3A4^62–65^. The elevated CYP interaction profiles of DMF and TMF are similarly consistent with the known behavior of methoxylated flavonoids, whose lipophilicity and aromatic methoxy substitutions facilitate interaction with hydrophobic catalytic regions of CYP enzymes^66–70^.

Collectively, the metabolic profiles of the Testolift phytochemicals support complementary pharmacokinetic behavior within the formulation. Protodioscin exhibited properties compatible with peripheral or extracellular target interaction, whereas diosgenin and the polymethoxyflavones demonstrated metabolic characteristics associated with intracellular accessibility and systemic distribution^27,53,54,71^. These coordinated metabolic features are consistent with the systems-oriented design of the Testolift formulation and its proposed multi-target interaction framework.

**Table 3:** In silico ADMET-AI predicted CYP450 inhibition and substrate liabilities of Testolift bioactive compounds.

Property	Protodioscin	Diosgenin	DMF	TMF
CYP1A2 inhibition	8.93 × 10^− 4^	4.55 × 10^− 3^	1	0.98
CYP2C19 inhibition	0.03	0.12	0.91	0.85
CYP2C9 inhibition	0.01	0.05	0.57	0.37
CYP2D6 inhibition	0.03	0.03	0.18	0.16
CYP3A4 inhibition	0.03	0.16	0.75	0.89
CYP2C9 substrate	0.01	0.06	0.25	0.25
CYP2D6 substrate	0.03	0.15	0.47	0.46
CYP3A4 substrate	0.52	0.69	0.59	0.64

### Distribution and excretion characteristics supporting the systemic and intracellular bioactivity of Testolift bioactives

The ADMET-AI distribution and excretion analysis revealed distinct pharmacokinetic behaviors among protodioscin, diosgenin, DMF, and TMF, reflecting differences in their physicochemical properties and molecular architectures (Table 4). Protodioscin exhibited the lowest predicted blood–brain barrier (BBB) penetration (0.25), moderate plasma protein binding (86.84%), negligible volume of distribution, and the longest predicted half-life (50.58 h), together with comparatively low microsomal clearance. In contrast, diosgenin, DMF, and TMF demonstrated higher BBB penetration probabilities and elevated plasma protein binding, consistent with the increased lipophilicity of steroidal sapogenins and polymethoxyflavones. Diosgenin also displayed a comparatively higher volume of distribution, whereas DMF and TMF exhibited moderate half-lives together with higher predicted hepatocyte and microsomal clearance values. These pharmacokinetic differences align with the structural characteristics of the investigated phytochemicals. The limited tissue distribution and prolonged systemic persistence of protodioscin are consistent with the behavior of highly polar glycosylated saponins, which are generally associated with extracellular or membrane-associated interactions^52,58,72^. In contrast, the lipophilic steroidal structure of diosgenin supports broader tissue distribution and intracellular accessibility within steroidogenic environments^73–75^. Similarly, the polymethoxyflavones DMF and TMF demonstrated distribution and clearance characteristics compatible with efficient membrane permeation and intracellular signaling-associated activity^76–79^. Collectively, the distribution and excretion profiles of the Testolift phytochemicals support complementary pharmacokinetic behavior within the formulation. Protodioscin exhibited characteristics compatible with peripheral target interaction, whereas diosgenin and the polymethoxyflavones demonstrated properties associated with intracellular accessibility and tissue-associated signaling activity. These coordinated pharmacokinetic features further support the systems-oriented multi-target framework proposed for the Testolift formulation, involving modulation of testosterone regulation, muscle physiology, and oxygen-dependent metabolic adaptation.

**Table 4:** In silico ADMET-AI predicted distribution and excretion profiles of Testolift phytochemical constituents.

Property	Protodioscin	Diosgenin	DMF	TMF
Blood-brain barrier penetration	0.25	0.84	0.87	0.79
Plasma protein binding rate (%)	86.84	100	96.35	91.94
Volume of distribution of steady state (VDss) (L/kg)	0.00	1.26	0	0
Half life (h)	50.58	16.88	14.25	17.51
Drug clearance (hepatocye) (mL/min/kg)	67.95	77.6	94.51	86.89
Drug clearance (microsome) (mL/min/kg)	21.15	33.15	45.77	38.33

### Toxicological profile and receptor interaction landscape of Testolift bioactive phytochemicals

The ADMET-AI toxicity and receptor interaction analysis revealed distinct safety and biological interaction profiles among the Testolift phytochemicals (Table 5). Predicted hERG inhibition probabilities were comparable across all compounds, while clinical toxicity, mutagenicity, and carcinogenicity predictions remained generally low, supporting a favorable overall toxicological profile. The moderate and comparable predicted hERG interaction probabilities observed across the investigated phytochemicals are consistent with previously reported interaction patterns of natural steroidal and flavonoid scaffolds, which may engage cardiac ion channels without necessarily producing clinically significant cardiotoxicity under physiological conditions^80–82^. Drug-induced liver injury (DILI) probabilities were comparatively higher for diosgenin and TMF, whereas protodioscin and DMF demonstrated lower predicted hepatic liability. Acute toxicity and skin reaction probabilities remained within moderate ranges across all investigated phytochemicals. The comparatively lower acute toxicity predictions associated with the polymethoxyflavones are consistent with previous reports describing the generally favorable toxicological characteristics of methoxyflavone-based phytochemicals^83^.

Receptor interaction predictions further reflected the structural diversity of the compounds. Diosgenin exhibited the highest predicted interaction probability with the full-length androgen receptor (AR-FL), consistent with previously reported androgen-supportive and steroidogenic characteristics of steroidal sapogenins^84^. DMF and TMF demonstrated comparatively greater predicted interactions with aromatase-related pathways and the aryl hydrocarbon receptor (AHR), whereas all compounds exhibited minimal interaction probabilities toward the androgen receptor ligand-binding domain (AR-LBD) and PPAR-γ. The uniformly low PPAR-γ interaction values suggest that the biological activities of the Testolift phytochemicals are unlikely to involve adipogenic or insulin-sensitizing nuclear receptor pathways.

Among transcription factor-related endpoints, DMF, TMF, and diosgenin demonstrated comparatively higher Nrf2/ARE activation probabilities, supporting potential antioxidant and cytoprotective signaling-associated effects. Predicted interactions associated with mitochondrial membrane potential and stress-response pathways remained within low-to-moderate ranges, while p53-related interaction probabilities were consistently low, indicating minimal predicted genotoxic stress.

Collectively, the toxicity and receptor interaction profiles support a favorable safety landscape together with biologically relevant multi-target interaction characteristics for the Testolift phytochemicals. Protodioscin exhibited properties compatible with peripheral or membrane-associated interaction behavior, diosgenin demonstrated characteristics associated with steroidogenic and androgen-related signaling, and the polymethoxyflavones exhibited interaction profiles consistent with intracellular signaling and antioxidant-associated pathways^53,54,71,85–88^. These coordinated biological interaction patterns further support the systems-oriented multi-target framework proposed for the Testolift formulation while maintaining an overall favorable predicted safety profile^50,57,61,72–94^.

**Table 5:** In silico ADMET-AI toxicity and receptor interaction predictions for Testolift constituents.

Property	Protodioscin	Diosgenin	DMF	TMF
hERG blocking	0.68	0.67	0.68	0.72
Clinical toxicity	0.09	0.08	0.02	0.03
Mutagenicity	0.31	0.27	0.4	0.36
Drug induced liver injury (DILI)	0.11	0.16	0.92	0.93
Carcinogenicity	0.01	0.02	0.04	0.05
Acute toxicity	3.62	2.61	2.59	2.53
Skin reaction	0.34	0.44	0.38	0.27
Androgen receptor (full length) (AR-FL)	0.11	0.24	0.11	0.13
Androgen receptor (ligand binding domain) (AR-LBD)	0.01	0.02	0.02	0.04
Aryl hydrocarbon receptor (AHR)	0.01	0.02	0.88	0.85
Aromatase	0.09	0.19	0.37	0.33
Estrogen receptor (full length) (ER-FL)	0.39	0.32	0.47	0.32
Estrogen receptor (ligand binding domain) (ER-LBD)	0.20	0.07	0.12	0.09
Peroxisome proliferator-activated receptor gamma (PPAR-γ)	0.01	0.01	0.03	0.02
Nuclear factor (erythroid- derived 2)-like 2/antioxidant responsive element (Nrf2/ARE)	0.24	0.44	0.57	0.52
ATPase family AAA domain containing protein 5 (ATAD5)	0.08	0.01	0.47	0.39
Heat shock factor (HSF) response element	0.02	0.11	0.06	0
Mitochondrial membrane potential	0.27	0.45	0.42	0.3
Tumor protein p53	0.08	0.02	0.08	0.1

### Molecular docking interactions of Testolift constituents with CYP19A1 (Aromatase)

**Fig. 2:**
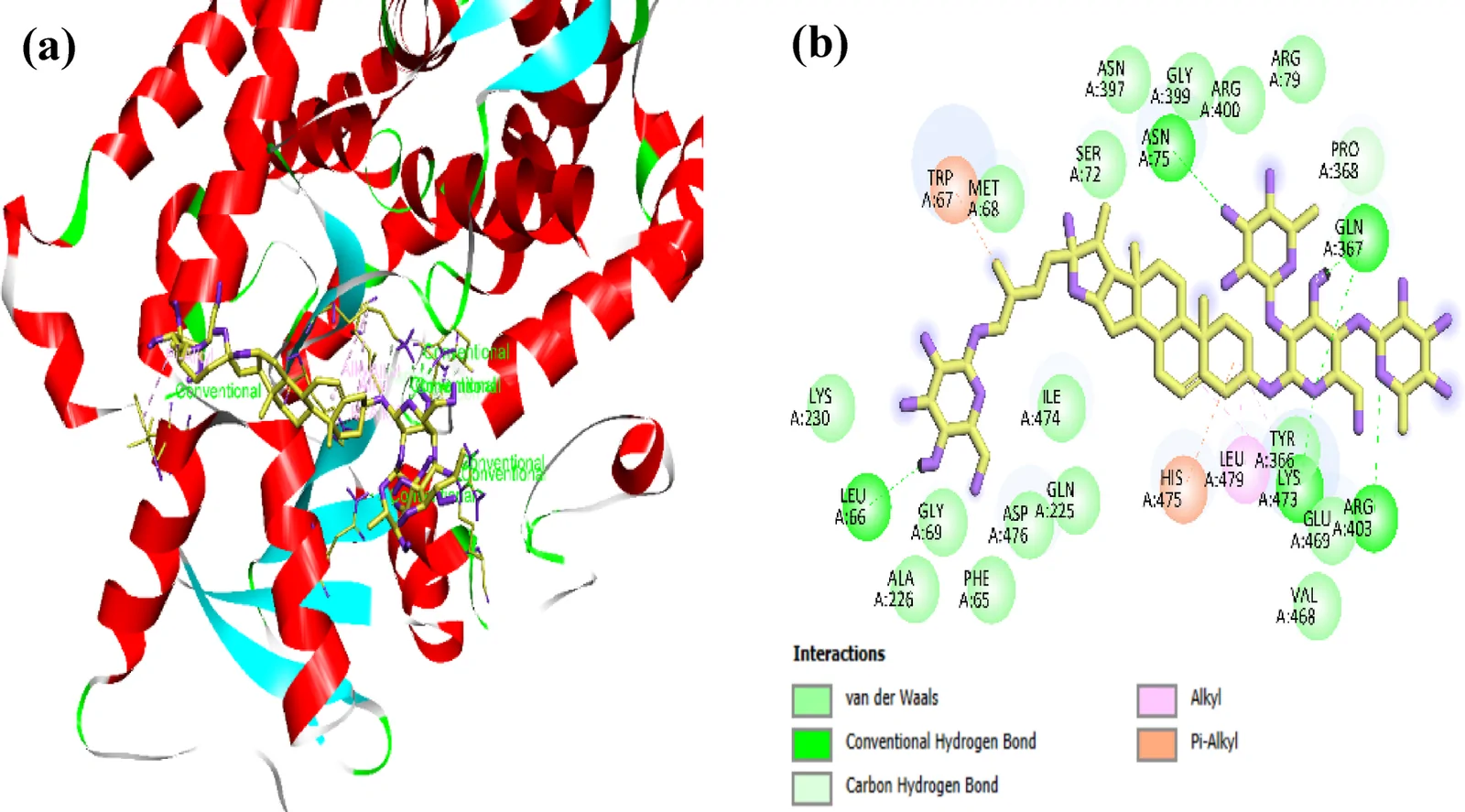
Three-dimensional (3D) and two-dimensional (2D) molecular docking interaction profiles of the Protodioscin–CYP19A1 complex. (**a**) Three-dimensional binding orientation of protodioscin within the CYP19A1 catalytic pocket showing hydrogen-bonding and residue interaction architecture. (**b**) Two-dimensional interaction map illustrating conventional hydrogen bonds, carbon hydrogen bonds, van der Waals interactions, alkyl interactions, and π–alkyl contacts involved in stabilization of the ligand within the active site of CYP19A1.

**Fig. 3:**
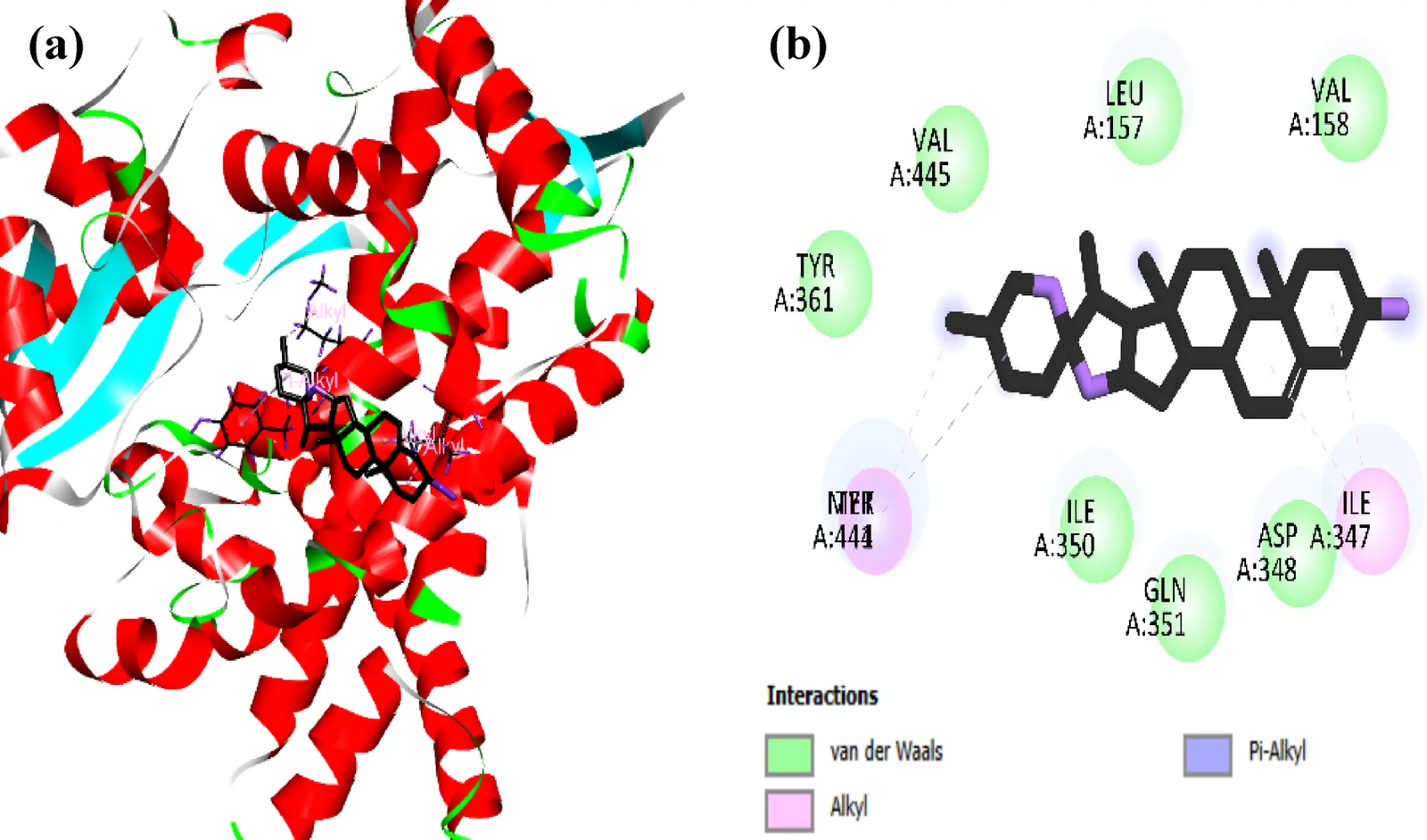
Three-dimensional (3D) and two-dimensional (2D) molecular docking interaction profiles of the Diosgenin–CYP19A1 complex. (**a**) Three-dimensional binding orientation of diosgenin within the CYP19A1 binding cavity illustrating hydrophobic interaction architecture and ligand accommodation within the protein environment. (**b**) Two-dimensional interaction map demonstrating predominant stabilization through alkyl, π–alkyl, and van der Waals interactions contributing to hydrophobic binding of diosgenin within the CYP19A1 active region.

**Fig. 4:**
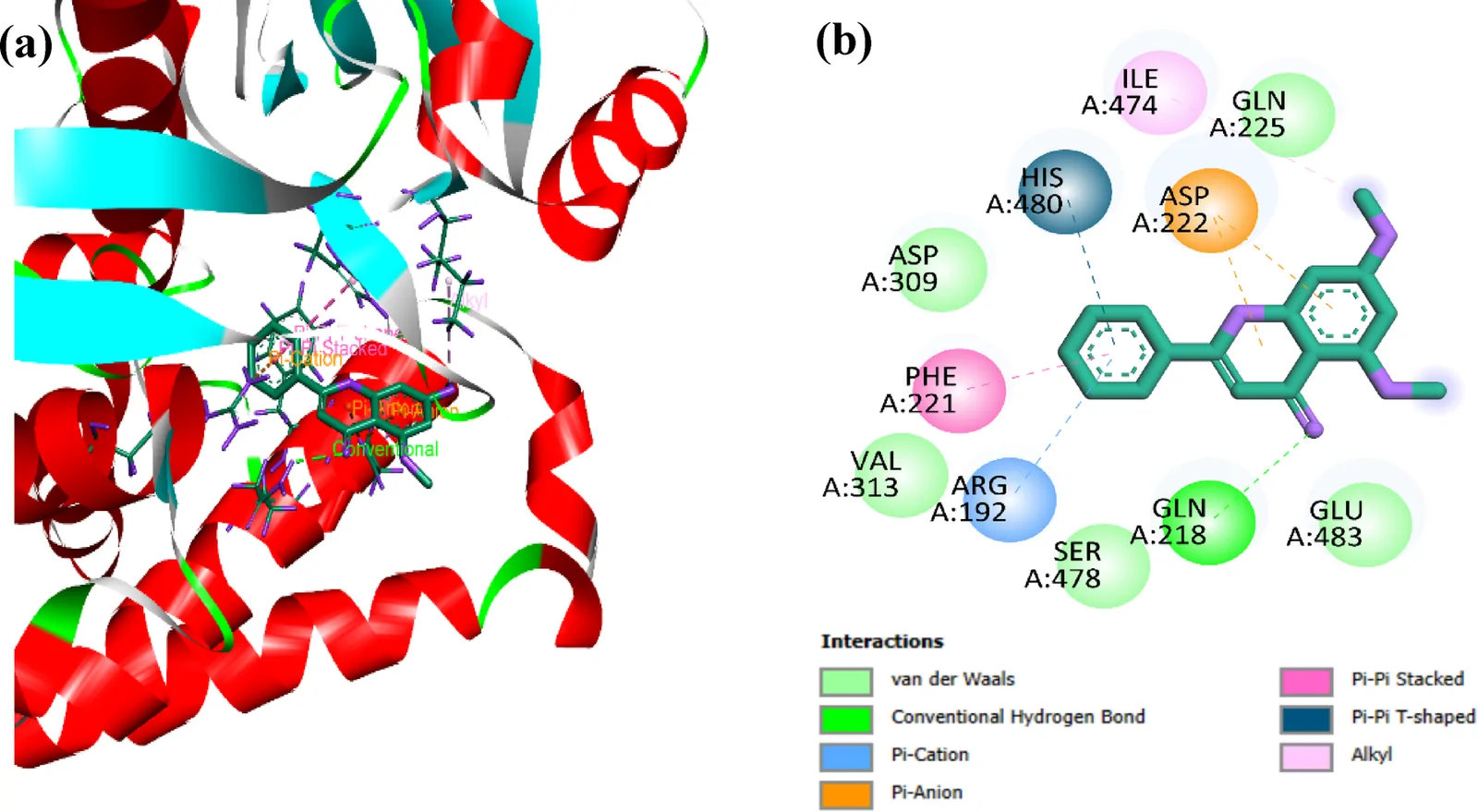
Three-dimensional (3D) and two-dimensional (2D) molecular docking interaction profiles of the 5,7-dimethoxyflavone (DMF)–CYP19A1 complex. (**a**) Three-dimensional binding orientation of DMF within the CYP19A1 catalytic cavity illustrating aromatic interaction architecture and ligand stabilization within the active site. (**b**) Two-dimensional interaction map revealing π–π stacking, π–cation, π–anion, hydrogen-bonding, and van der Waals interactions associated with stabilization of DMF within the CYP19A1 binding pocket.

**Fig. 5:**
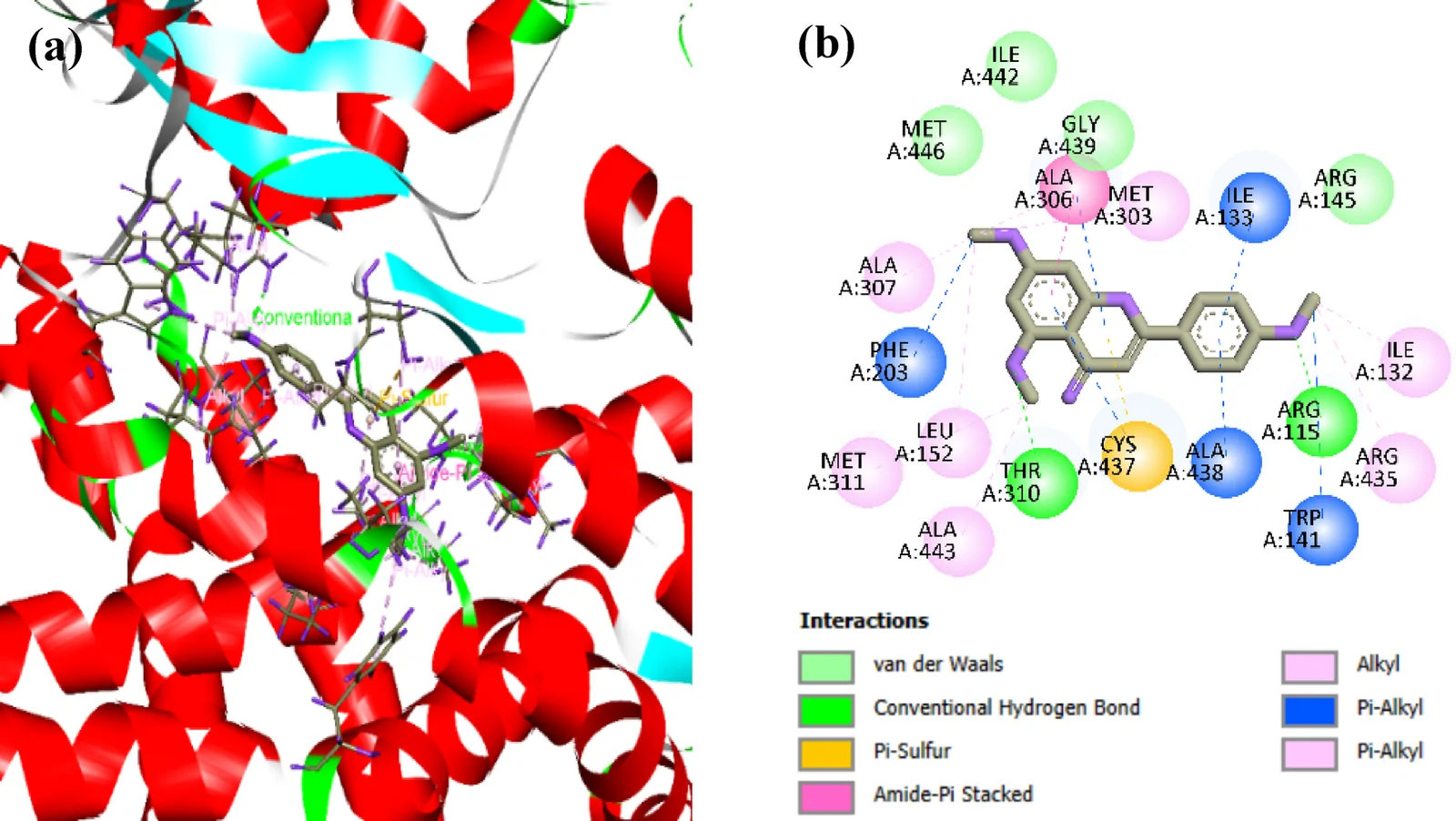
Three-dimensional (3D) and two-dimensional (2D) molecular docking interaction profiles of the 5,7,4′-trimethoxyflavone (TMF)–CYP19A1 complex. (**a**) Three-dimensional binding orientation of TMF within the CYP19A1 active-site cavity showing ligand accommodation and interaction geometry. (**b**) Two-dimensional interaction map illustrating π–alkyl, π–sulfur, amide–π stacking, hydrogen-bonding, and van der Waals interactions contributing to stabilization of TMF within the CYP19A1 catalytic region.

The docking analysis of the CYP19A1 active site with the four principal Testolift bioactive compounds such as protodioscin, diosgenin, DMF, and TMF revealed distinct and mechanistically meaningful binding patterns that reflect their structural diversity and potential to modulate aromatase activity through complementary interaction mechanisms (Figs. 2, 3, 4 and 5).

Protodioscin exhibited the most extensive interaction network, engaging the CYP19A1 catalytic pocket through a dense array of polar, hydrophobic, and dispersion contacts (Fig. 2a, b). The ligand was strongly anchored by multiple conventional hydrogen bonds formed with Leu66, Asn75, Gln367, Lys473, and Arg403, collectively stabilizing its orientation toward the active center. Additional carbon–hydrogen bonding interactions with Pro368 and Tyr366 contributed to ligand stabilization along the entrance of the pocket. Protodioscin further established π–alkyl interactions with Trp67 and His475, alongside alkyl contacts involving Leu479, Lys473, and Tyr366, which supported its steric accommodation. Surrounding these interactions was an extensive shell of van der Waals contacts with residues such as Lys230, Ala226, Gly69, Phe65, Asp470, Gln225, Ile474, Glu469, Val468, Arg79, Arg400, Gly399, Asn397, Ser72, and Met68. The combination of strong polar anchoring and widespread dispersion contacts is consistent with the known tendency of large steroidal saponins to modulate aromatase by occupying and sterically hindering access to the catalytic cavity, thereby reducing estradiol formation without relying on classic active-site blockade^58,95,96^.

Diosgenin displayed a markedly different binding profile characterized by predominately hydrophobic interactions (Fig. 3a, b). The steroidal aglycone inserted deeply into the hydrophobic regions of the active site, forming strong alkyl interactions with Met444 and a pair of alkyl contacts with Ile347. A π–alkyl interaction with Tyr441 further supported the stability of the ligand within the lipophilic core. Diosgenin was additionally stabilized through van der Waals interactions with Tyr361, Val445, Leu157, Val158, Ile350, Gln351, and Asp348. This hydrophobic mode of engagement reflects the natural affinity of the steroidal skeleton of the diosgenin for lipid-rich microenvironments and supports earlier observations that diosgenin modulates aromatase activity and the androgen-estrogen axis through nonpolar interactions within P450 catalytic surfaces^26,47,64,97^.

The polymethoxyflavone, DMF demonstrated an interaction pattern dominated by aromatic and charged contacts, consistent with its planar phenylchromenone structure (Fig. 4a, b). A well-oriented conventional hydrogen bond with Gln218 provided a key directional anchor. This was complemented by two distinct π–anion interactions with Asp222, which helped stabilize the aromatic nucleus in a negatively charged pocket. DMF also engaged in a π–cation interaction with Arg192 and formed π–π stacked interactions with Phe221, while a T-shaped π–π interaction with His480 further reinforced ligand positioning. Additionally, the compound interacted through alkyl contacts with Ile474 and established van der Waals interactions with Asp309, Val313, Ser478, Glu483, Thr484, and Gln225. The presence of multiple aromatic interactions is consistent with experimental evidence showing that methoxylated flavones inhibit aromatase by inserting their planar aromatic scaffolds into π-rich active-site regions^98–101^.

TMF demonstrated the most diverse interaction profile among the four compounds (Fig. 5a, b). Its orientation was stabilized by conventional hydrogen bonds with Thr310 and Arg115. TMF formed extensive alkyl contacts with Arg435, Ile132, Met303, Ala306, Ala307, Leu252, Met311, and Ala343. A second set of interactions involved π–alkyl contacts with Ile133, Ala438, Trp141, Cys437, Ala306, and Phe205. Notably, TMF exhibited an amide–π stacked interaction with Ala306 and a π–sulfur interaction with Cys437, reflecting a strong complementarity between the methoxylated aromatic core and sulfur-containing residues. Additional van der Waals interactions with Met446, Ile442, Gly439, and Arg145 reinforced the overall binding conformation. These findings mirror structural studies showing that polymethoxyflavones exert aromatase inhibitory activity through a blend of hydrophobic and π-driven interactions that collectively stabilize ligand placement across the catalytic site^28,53,54,98–101^.

Overall, these results reveal a multi-target interaction plausibility strategy that strengthens the mechanistic foundation of the Testolift design. Protodioscin functions through extensive polar anchoring and steric interference, creating a broad modulation of substrate access. Diosgenin exerts its effects through deep hydrophobic insertion, consistent with its role in balancing androgen and estrogen levels via steroidogenic interactions. DMF and TMF act through aromatic stacking, charged interactions, and mixed hydrophobic contacts mechanisms characteristic of flavone-based aromatase inhibitors. The structural complementarity of these interactions provides strong molecular support for the capacity of the formulation to attenuate excessive testosterone-to-estradiol conversion through distinct yet synergistic binding modes at the CYP19A1 active site.

### Molecular docking interactions of Testolift bioactives with myostatin (GDF-8)

**Fig. 6:**
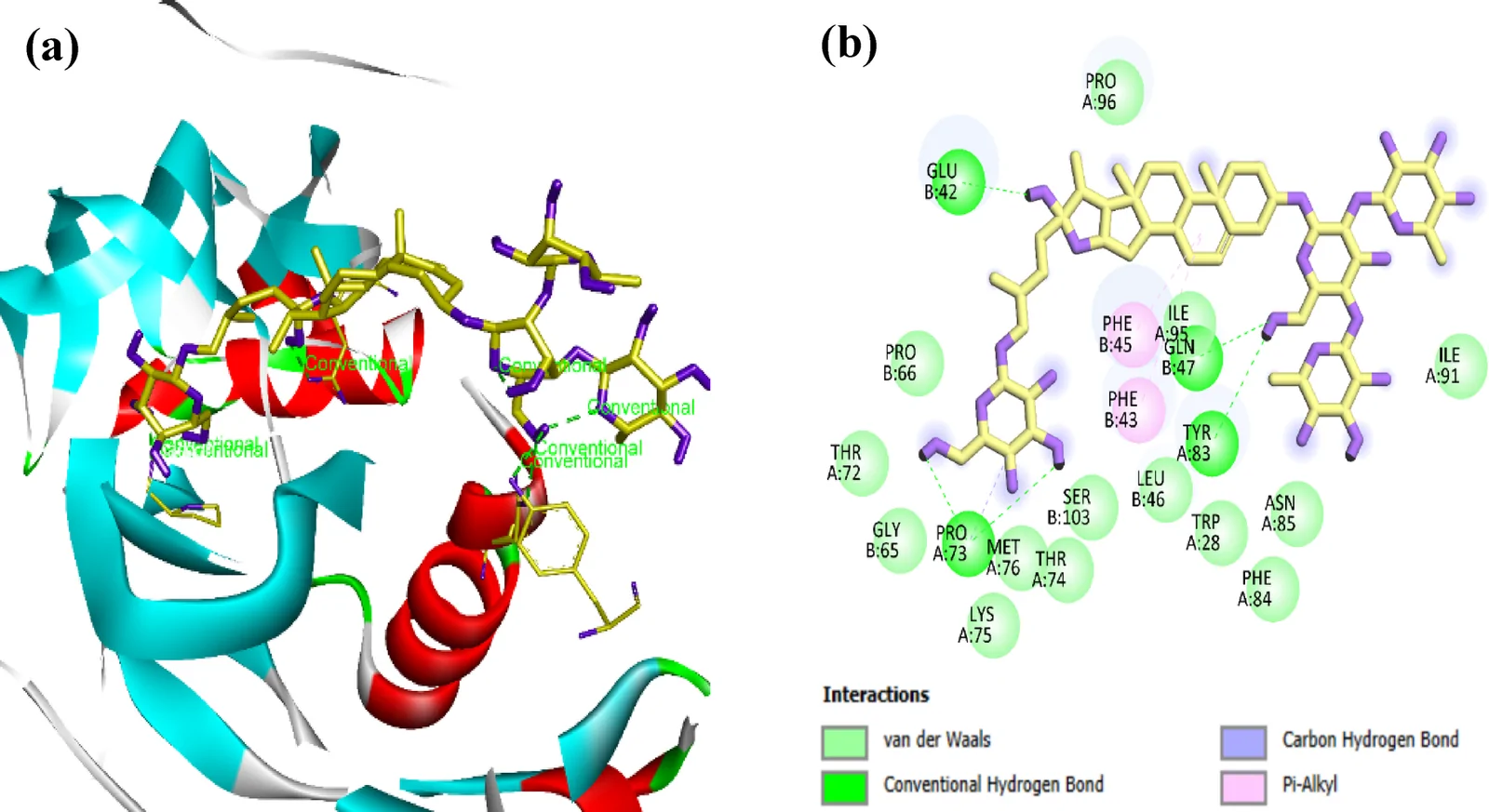
Three-dimensional (3D) and two-dimensional (2D) molecular docking interaction profiles of the Protodioscin–Myostatin (GDF-8) complex. (**a**) Three-dimensional binding orientation of protodioscin within the Myostatin ligand-binding cleft illustrating extensive interaction architecture and ligand accommodation. (**b**) Two-dimensional interaction map showing hydrogen bonding, carbon–hydrogen bonding, van der Waals interactions, and π–alkyl contacts contributing to stabilization of protodioscin within the Myostatin inhibitory region.

**Fig. 7:**
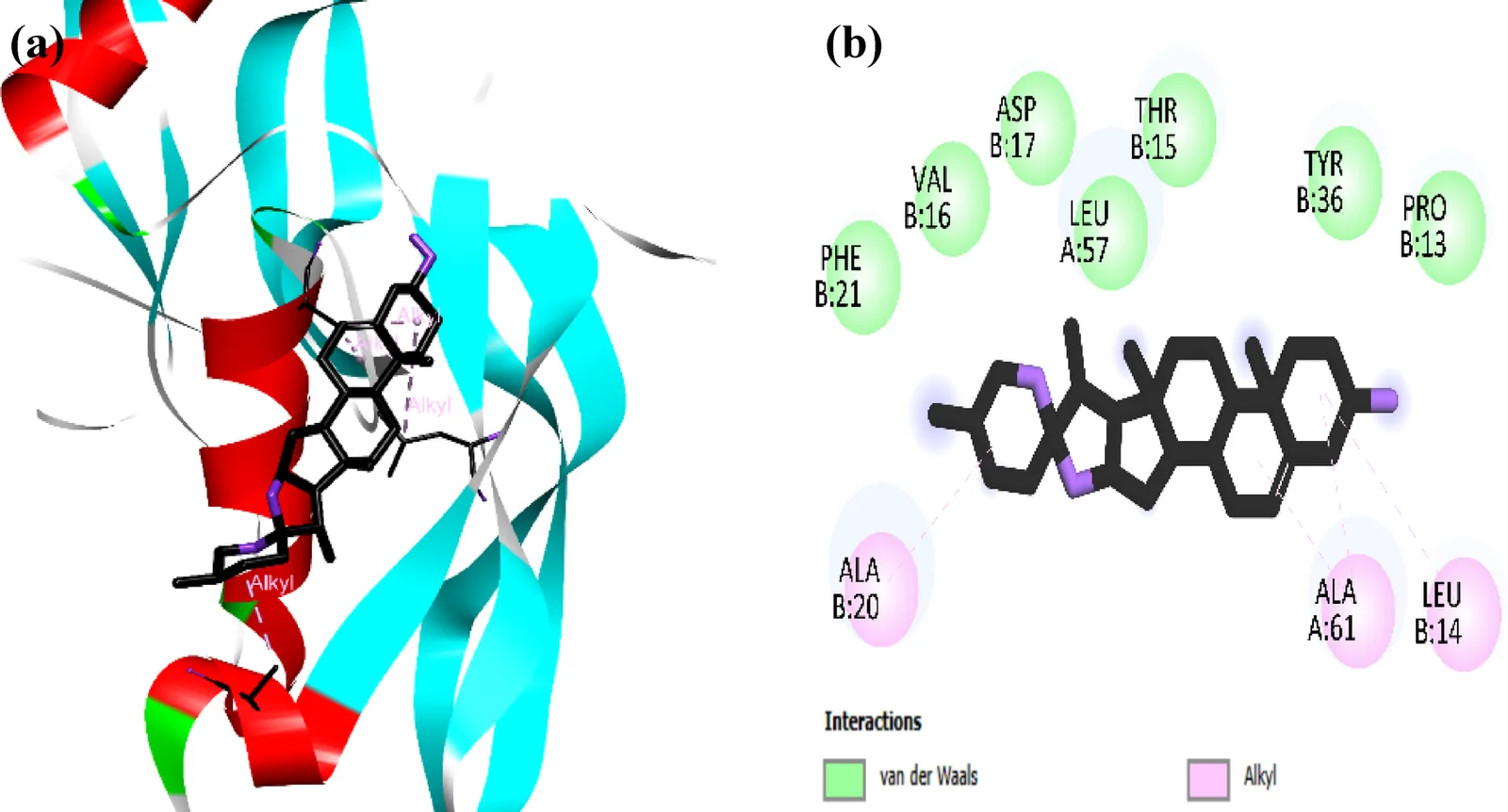
Three-dimensional (3D) and two-dimensional (2D) molecular docking interaction profiles of the Diosgenin–Myostatin (GDF-8) complex. (**a**) Three-dimensional binding orientation of diosgenin within the Myostatin receptor pocket illustrating hydrophobic interaction architecture. (**b**) Two-dimensional interaction map demonstrating predominant stabilization through alkyl, π–alkyl, and van der Waals interactions associated with hydrophobic ligand binding within the Myostatin active interface.

**Fig. 8:**
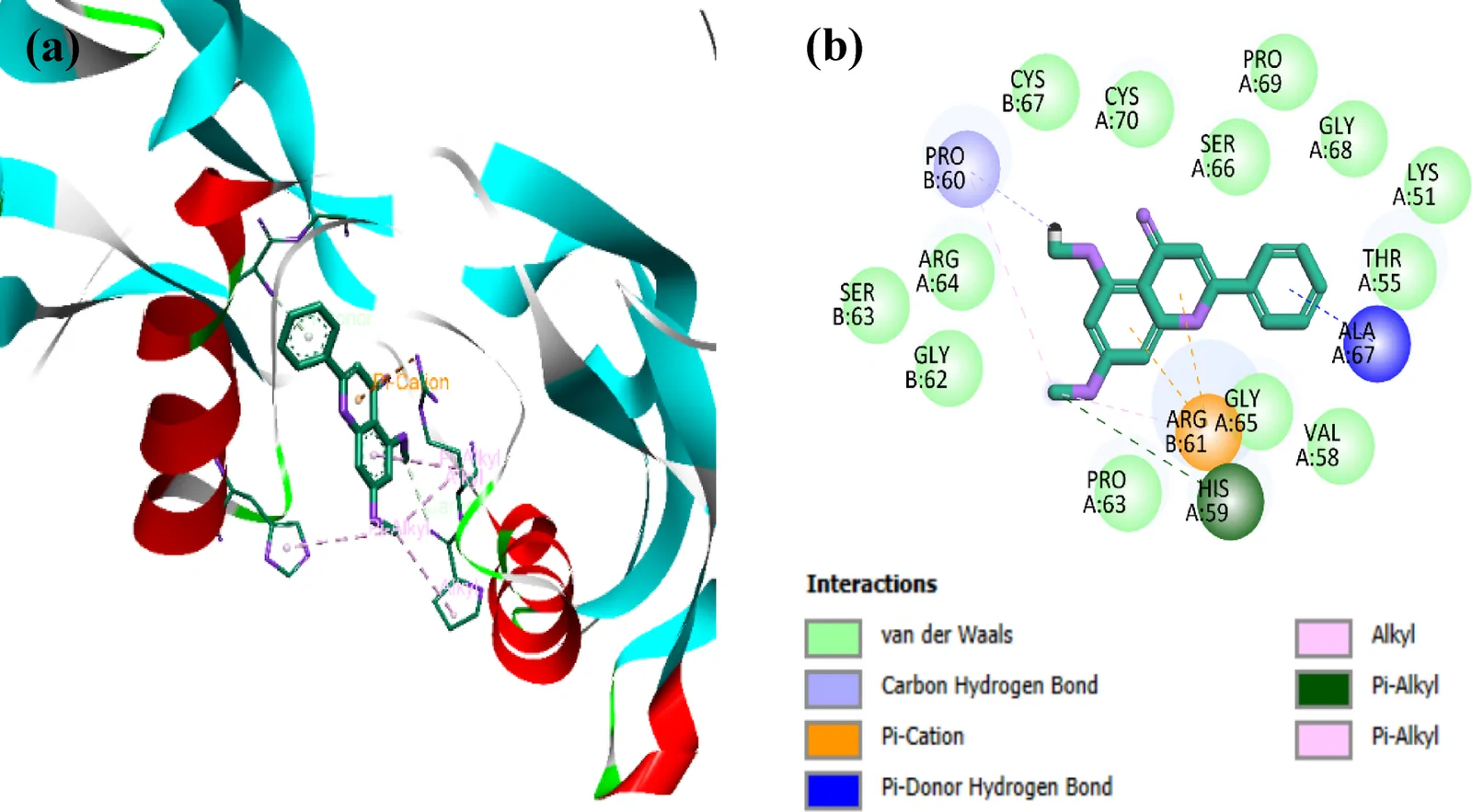
Three-dimensional (3D) and two-dimensional (2D) molecular docking interaction profiles of the 5,7-dimethoxyflavone (DMF)–Myostatin (GDF-8) complex. (**a**) Three-dimensional binding orientation of DMF within the Myostatin interaction cavity illustrating aromatic interaction geometry and ligand stabilization. (**b**) Two-dimensional interaction map demonstrating π–alkyl, π–cation, π-donor hydrogen bonding, carbon–hydrogen bonding, and van der Waals interactions involved in stabilization of DMF within the receptor interface.

**Fig. 9:**
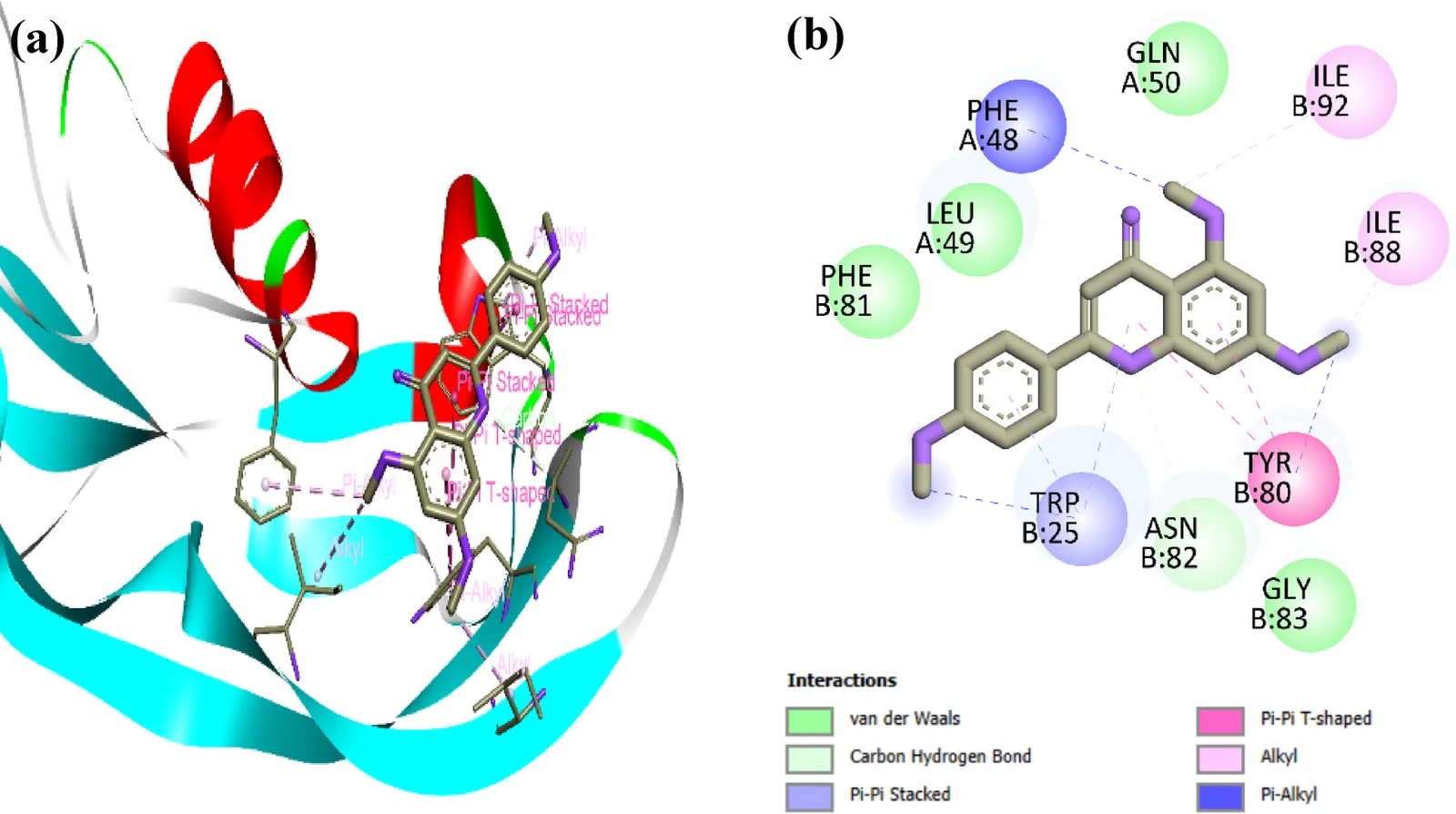
Three-dimensional (3D) and two-dimensional (2D) molecular docking interaction profiles of the 5,7,4′-trimethoxyflavone (TMF)–Myostatin (GDF-8) complex. (**a**) Three-dimensional binding orientation of TMF within the Myostatin inhibitory region showing ligand accommodation and interaction geometry. (**b**) Two-dimensional interaction map highlighting π–π stacking, π–π T-shaped interactions, π–alkyl stabilization, hydrogen bonding, and van der Waals interactions contributing to stabilization of TMF within the Myostatin binding pocket.

The binding interactions of the Testolift constituents with Myostatin revealed structurally diverse and mechanistically complementary inhibitory profiles, reflecting the distinct chemical architectures of steroidal saponins, steroidal sapogenins, and polymethoxyflavones (Figs. 6, 7, 8 and 9). Protodioscin demonstrated a rich interaction landscape within the Myostatin binding groove (Fig. 6a, b). The ligand was anchored through a conventional hydrogen bond network involving Pro73, Glu42, Gln47, and Tyr83, while an additional carbon-hydrogen bond with Pro73 contributed to its stabilizing interactions at the pocket entrance. The compound further formed π–alkyl interactions with Phe45 and Phe43, complementing its amphipathic architecture. This configuration was further supported by widespread van der Waals contacts involving Pro66, Thr72, Gly65, Met76, Thr74, Lys75, Ser103, Ile95, Leu46, Trp28, Asn85, Pro96, Ile91, and Phe91. The convergence of polar anchoring and hydrophobic dispersion forces allowed protodioscin to occupy a broad segment of the Myostatin inhibitory cleft, a mode of interaction consistent with the known capacity of steroidal saponins to disrupt protein-protein interactions across large surface areas of regulatory cytokines such as Myostatin^50,102,103^.

Diosgenin exhibited a contrasting yet equally relevant binding mode, primarily governed by hydrophobic interactions (Fig. 7a, b). Alkyl contacts with Ala20, Ala61, and Leu14 created a stable nonpolar scaffold within the Myostatin surface. These interactions were further surrounded by van der Waals contributions involving Phe21, Val16, Asp17, Leu57, Thr15, Tyr36, and Pro13. The overall hydrophobic engagement is fully consistent with the lipophilic steroidal backbone of diosgenin, which allows it to target hydrophobic hot spots on protein surfaces implicated in Myostatin activation and receptor binding^104–107^. Predicted binding pose of diosgenin suggests that it may interfere with Myostatin maturation or receptor docking, thereby complementing its well-reported anabolic and anti-catabolic biological effects.

The DMF bound Myostatin through a combination of aromatic and polar interactions (Fig. 8a, b). Alkyl contacts with Arg61 and Pro60 initiated hydrophobic stabilization, while π–alkyl interactions involving His59 and Arg61 highlighted the aromatic complementarity of the flavone scaffold. DMF also formed a carbon–hydrogen bond with Pro60, a π–cation interaction with Arg61, and a π-donor hydrogen bond with Ala67. These interactions allowed DMF to fit snugly within the Myostatin cleft, with its aromatic ring system forming directed interactions with side chains implicated in substrate–protein recognition. This binding configuration is consistent with prior observations that methoxylated flavones inhibit pro-inflammatory and catabolic signaling proteins by stabilizing allosteric cavities or interfering with ligand-binding surfaces^108–112^.

TMF demonstrated the most structurally intricate interaction pattern (Fig. 9a, b). A key carbon–hydrogen bond with Asn82 provided polar anchoring, while an array of hydrophobic contacts with Ile92 and Ile88 established a strong nonpolar base for ligand stabilization. Aromatic system of the TMF formed several π–alkyl interactions with Phe48, Tyr80, and Trp25, supplemented by dual π–π T-shaped interactions with Tyr80. Additionally, the ligand formed a π–π stacked interaction with Trp25, creating a highly stabilized aromatic sandwich within the Myostatin pocket. Numerous van der Waals interactions involving Gly83, Phe81, Leu49, and Gln50 further solidified this binding mode. These outcomes mirror structural pharmacology findings showing that polymethoxyflavones possess high affinity for planar or semi-planar binding clefts within regulatory proteins such as Myostatin, enabling them to disrupt ligand–receptor interfaces involved in muscle catabolism.

Collectively, these docking results indicate that the Testolift formulation engages Myostatin through multiple distinct molecular mechanisms: protodioscin via dual polar–hydrophobic anchoring, diosgenin through compact hydrophobic insertion, and DMF/TMF through sophisticated π-driven aromatic interactions. This complementary binding diversity enhances the potential of the formulation to attenuate Myostatin activity, thereby supporting muscle growth, reducing catabolic signaling, and contributing to improved strength and endurance outcomes associated with elevated testosterone bioefficacy.

### Molecular docking interactions of Testolift bioactives with prolyl hydroxylase-2 (PHD2)

**Fig. 10:**
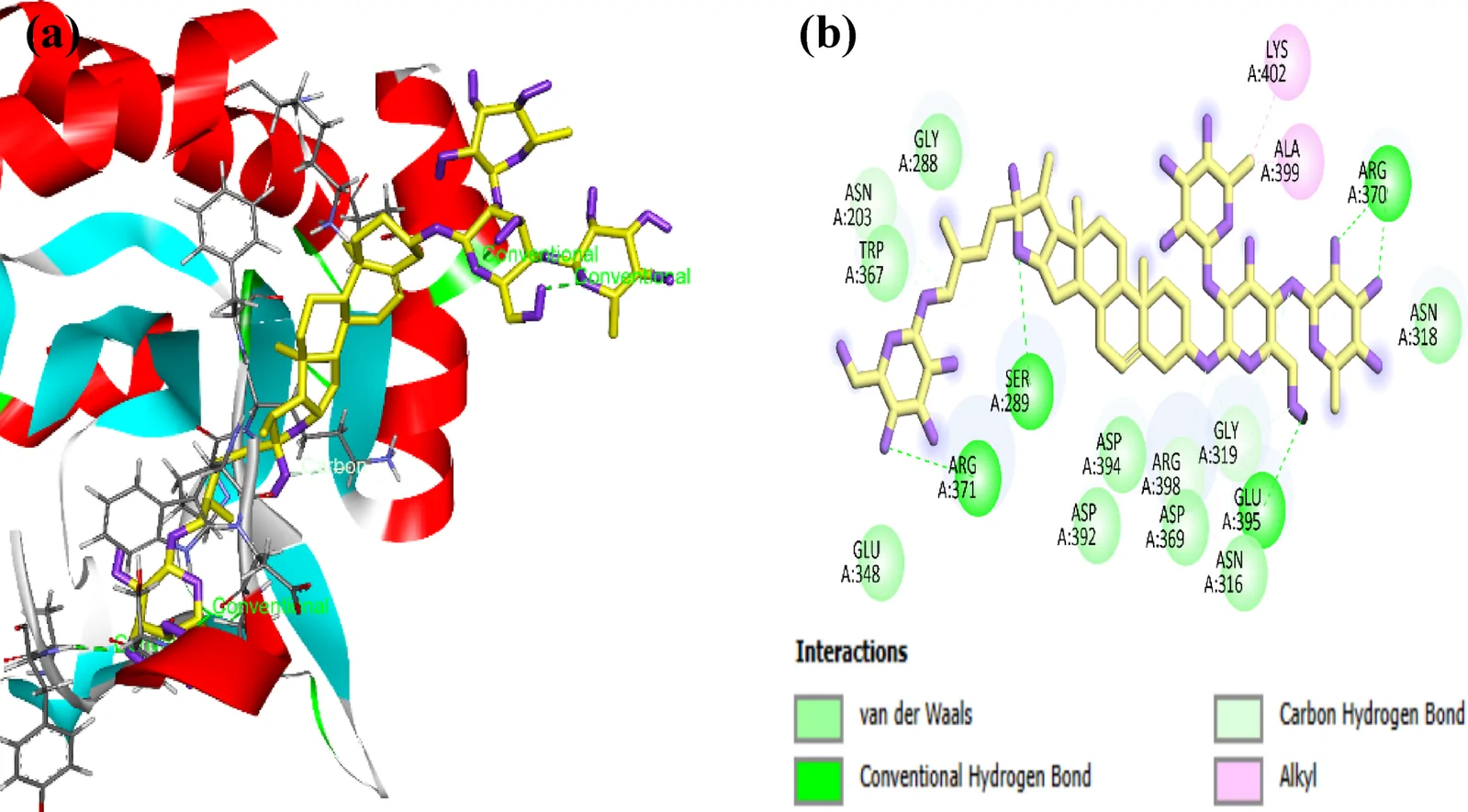
Three-dimensional (3D) and two-dimensional (2D) molecular docking interaction profiles of the Protodioscin–Prolyl Hydroxylase-2 (PHD2) complex. (**a**) Three-dimensional binding orientation of protodioscin within the PHD2 catalytic cavity illustrating extensive interaction architecture and ligand accommodation along the oxygen-sensing active cleft. (**b**) Two-dimensional interaction map showing multiple hydrogen bonds, carbon–hydrogen interactions, alkyl contacts, and extensive van der Waals interactions contributing to stabilization of protodioscin within the PHD2 binding region.

**Fig. 11:**
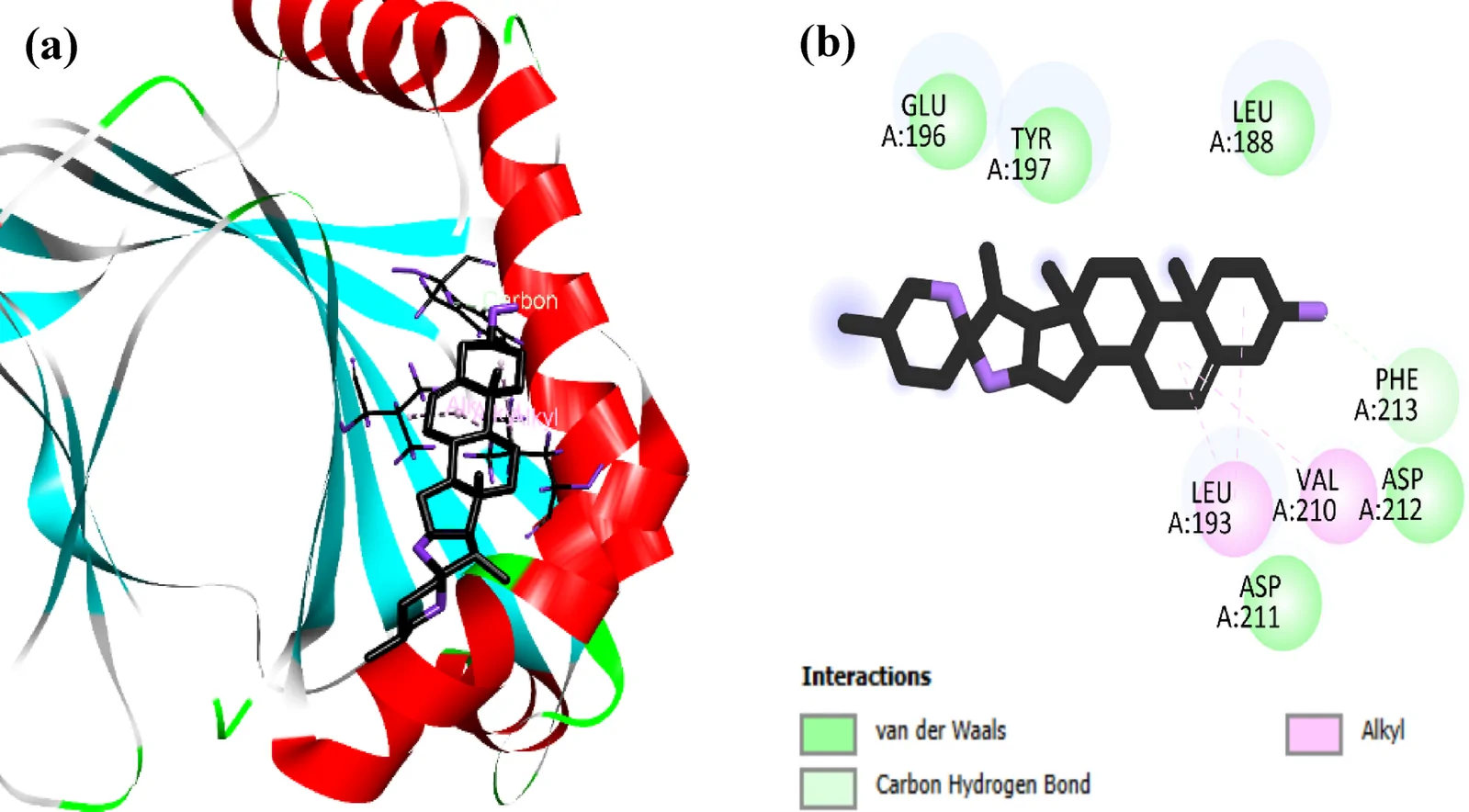
Three-dimensional (3D) and two-dimensional (2D) molecular docking interaction profiles of the Diosgenin–Prolyl Hydroxylase-2 (PHD2) complex. (**a**) Three-dimensional binding orientation of diosgenin within the PHD2 regulatory cavity illustrating hydrophobic insertion and ligand accommodation near catalytic residues. (**b**) Two-dimensional interaction map demonstrating hydrophobic stabilization, carbon–hydrogen interactions, alkyl interactions, and van der Waals contacts associated with diosgenin binding within the PHD2 active region.

**Fig. 12:**
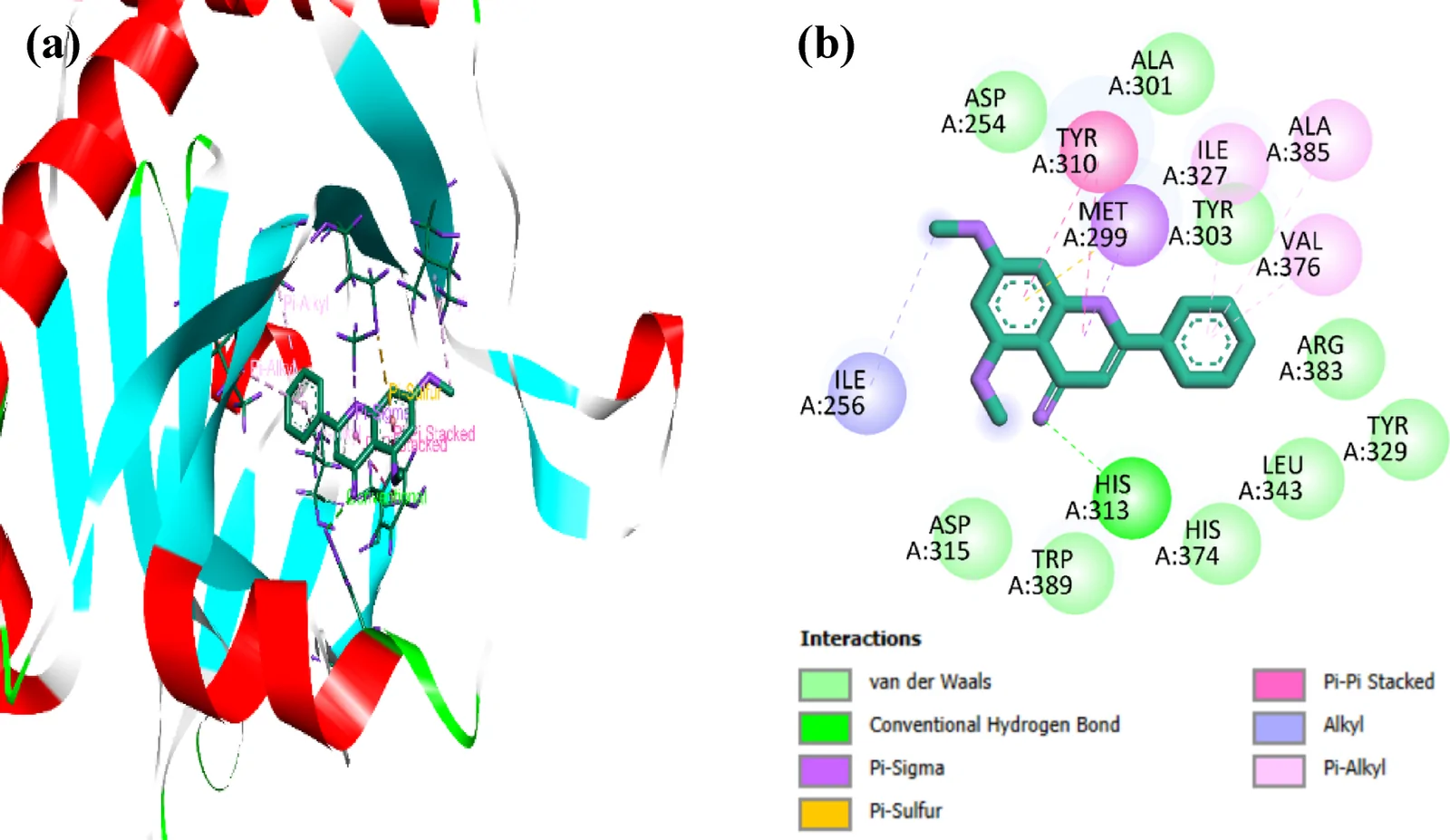
Three-dimensional (3D) and two-dimensional (2D) molecular docking interaction profiles of the 5,7-dimethoxyflavone (DMF)–Prolyl Hydroxylase-2 (PHD2) complex. (**a**) Three-dimensional binding orientation of DMF within the PHD2 interaction cavity illustrating aromatic interaction geometry and ligand stabilization. (**b**) Two-dimensional interaction map revealing π–π stacking, π–alkyl interactions, π-sulfur contacts, hydrogen bonding, and van der Waals interactions involved in stabilization of the DMF aromatic scaffold within the PHD2 binding pocket.

**Fig. 13:**
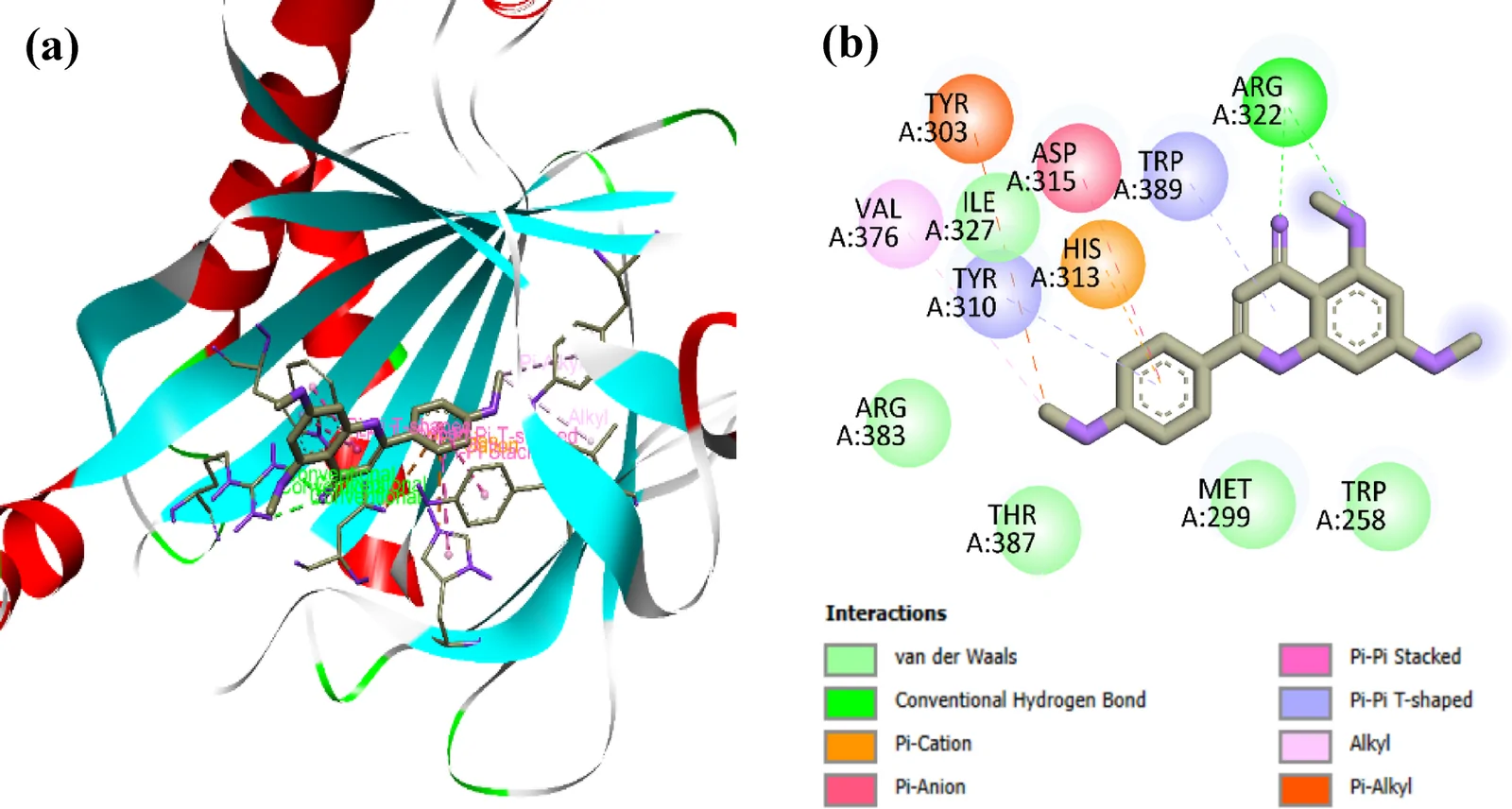
Three-dimensional (3D) and two-dimensional (2D) molecular docking interaction profiles of the 5,7,4′-trimethoxyflavone (TMF)–Prolyl Hydroxylase-2 (PHD2) complex. (**a**) Three-dimensional binding orientation of TMF within the HIF-regulatory region of PHD2 showing ligand accommodation and interaction geometry. (**b**) Two-dimensional interaction map illustrating π–π T-shaped stacking, π-anion, π-cation, π–alkyl, alkyl interactions, hydrogen bonding, and van der Waals interactions contributing to stabilization of TMF within the PHD2 catalytic environment.

The docking interactions of the Testolift constituents with Prolyl Hydroxylase-2 (PHD2) reveal a series of structurally complementary binding modes that are highly consistent with the intention of the formulation to modulate oxygen-sensing pathways and enhance mitochondrial activity, erythropoiesis, and endurance physiology (Figs. 10, 11, 12 and 13). PHD2 is the central oxygen sensor responsible for hydroxylation-dependent degradation of HIF-1α, and natural inhibitors frequently bind within the catalytic pocket to stabilize HIF signaling^113,114^.

Protodioscin demonstrated a rich and multidimensional interaction pattern within the PHD2 active site (Fig. 10a, b). The ligand was anchored by conventional hydrogen bonds with Arg371, Ser289, Arg370, and Glu395, providing strong polar stabilization near the catalytic center. These were complemented by carbon–hydrogen bonds involving Asn203, Gly319, and Arg398, positioning the molecule across the hydroxylation interface. Alkyl interactions with Lys402 and Ala399 supported its hydrophobic engagement. These strong interactions were embedded in a broad van der Waals network encompassing Glu348, Gly206, Lys204, His205, Met202, Gly288, Trp367, Asn318, Asn316, Asp369, Asp394, Asp392, Tyr290, and Gly206. This combination of polar anchoring and hydrophobic surface coverage allowed protodioscin to span the catalytic cavity, hindering access to essential oxygen and iron-binding residues required for HIF-1α hydroxylation. Such behavior is consistent with reported mechanisms of bulky steroidal saponins, which inhibit oxygen-sensing enzymes and promote HIF stabilization under normoxic conditions^114–118^.

Diosgenin adopted a hydrophobic mode of interaction, typical of steroidal sapogenins (Fig. 11a, b). Two strong alkyl contacts with Leu193, along with an additional alkyl interaction with Val210, supported its insertion into the hydrophobic regions of the enzyme. A carbon–hydrogen bond with Phe213 contributed additional stabilization. The ligand was surrounded by van der Waals interactions involving Glu196, Tyr197, Leu188, Asp212, and Asp211, forming a cohesive hydrophobic cluster. This mode of binding is consistent with the capacity of diosgenin to enhance mitochondrial function and erythropoietic signaling via modulation of oxygen-sensing pathways.

DMF exhibited a more aromatic and electronically diverse interaction pattern (Fig. 12a, b). A key hydrogen bond with His313 provided directional stabilization near the metal-binding region of PHD2. π–alkyl contacts with Ile327, Ala385, and Val376, together with a strong π–sulfur interaction with Met299, positioned the ligand within an aromatic docking region adjacent to the catalytic core. DMF also formed an alkyl contact with Ile256, while extensive van der Waals forces engaged Asp254, Ala301, Tyr303, Arg383, Tyr329, Leu343, His374, Trp389, and Asp315. The presence of dual π–π stacking interactions with Tyr310 suggests that DMF engages residues critical for conformational flexibility and catalytic function. These interactions align closely with the known behavior of polymethoxyflavones, which stabilize HIF-1α by interfering with PHD2-mediated hydroxylation.

TMF demonstrated the most sophisticated binding architecture among the flavonoids (Fig. 13a, b). Two conventional hydrogen bonds with Arg322 formed the primary anchoring points. This was complemented by π–alkyl interactions with Tyr303 and alkyl stabilization through Val376. TMF’s aromatic rings engaged in π–π T-shaped stacking with Tyr310 and Trp389, while π-anion and π-cation interactions with Asp315 and His313 respectively reinforced the electrostatic complementarity of the binding mode. Van der Waals contacts surrounding Ile327, Arg383, Thr387, Met299, and Trp258 further stabilized the ligand within the active pocket. This combination of π-driven interactions, hydrogen bonding, and hydrophobic engagement suggests that TMF has the capacity to effectively hinder PHD2 catalytic activity, thereby promoting HIF-1α stabilization and downstream mitochondrial and vascular responses^39,119–121^.

Together, these findings illustrate that the Testolift formulation targets PHD2 through multiple binding mechanisms. Protodioscin provides broad catalytic obstruction through an extensive polar and hydrophobic network; diosgenin engages tightly within hydrophobic clusters implicated in substrate recognition; and DMF and TMF use aromatic stacking, π–electron interactions, hydrogen bonding, and mixed polar–nonpolar contacts to occupy the HIF-regulatory site. This diversity of inhibitory modes reinforces the multi-targeted design logic of Testolift, positioning the formulation to enhance oxygen utilization, mitochondrial efficiency, and endurance performance through coordinated PHD2 modulation.

### Comparative molecular interaction profiling of Testolift bioactives across CYP19A1, myostatin, and prolyl hydroxylase-2

The comparative analysis of the molecular interaction patterns for protodioscin, diosgenin, 5,7-dimethoxyflavone (DMF), and 5,7,4′-trimethoxyflavone (TMF) across CYP19A1, myostatin, and prolyl hydroxylase-2 (PHD2) reveals a remarkable degree of structural complementarity among the four bioactive constituents (Table 6). This complementarity supports the mechanistic basis of the Testolift formulation, which is designed to influence testosterone preservation, myostatin suppression, and oxygen-dependent metabolic regulation through coordinated multi-ligand targeting.

Protodioscin exhibited the highest density of interactions across all three protein targets, characterized by substantial van der Waals contacts such as 15 in CYP19A1, 14 in myostatin, and 5 in PHD2. These extensive dispersion forces correspond to its bulky, flexible steroidal saponin architecture, which enables the molecule to occupy broad protein surfaces and interact with both catalytic and peripheral residues. The presence of numerous conventional hydrogen bonds in CYP19A1 (six bonds), myostatin (five bonds), and PHD2 (two bonds) further underscores the ability of the protodioscin to anchor itself through strong polar interactions. This anchoring is reinforced by additional carbon–hydrogen bonds, alkyl contacts, and π–alkyl interactions, which together create a multi-point binding mode capable of influencing enzymatic behavior through steric hindrance and polarity-driven stabilization. Such interaction patterns are characteristic of steroidal saponins that exhibit broad regulatory effects on endocrine and metabolic enzymes through extensive surface occupancy and polar engagement^47,50,58,95,96,114^. The extensive interaction network observed for protodioscin across CYP19A1, Myostatin, and PHD2 supports its potential role as a multi-target modulator within the Testolift formulation.

Although the predicted docking affinities among the Testolift phytochemicals were within a relatively narrow range, the interaction analyses revealed clear differences in binding architecture, including variations in hydrogen bonding, hydrophobic stabilization, aromatic stacking, π-driven interactions, and protein surface occupancy. These distinctions suggest coordinated rather than strictly selective modes of target engagement. Such behavior is consistent with multi-component nutraceutical systems, in which modulation of interconnected biological pathways may be more physiologically relevant than extremely high affinity toward a single isolated target.

Importantly, compounds such as protodioscin possess physicochemical properties that deviate from conventional small-molecule drug-likeness parameters due to their high molecular weight, glycosylation, and structural complexity. Consequently, lower QED values should not necessarily be interpreted as indicators of poor biological relevance. Natural products frequently exhibit unique topological and stereochemical features that enable biologically meaningful receptor interactions despite limited compliance with traditional drug-likeness filters. Feher and Schmidt^122^ reported that structurally complex natural products often maintain favorable receptor-binding characteristics despite deviating from conventional medicinal chemistry criteria. The broad interaction surface and extensive polar contact network observed for protodioscin across CYP19A1, Myostatin, and PHD2 are consistent with this established behavior of structurally complex steroidal saponins.

**Table 6:** Comparative quantitative analysis of molecular docking interaction profiles for protodioscin, diosgenin, 5,7-dimethoxyflavone (DMF), and 5,7,4′-trimethoxyflavone (TMF) across CYP19A1 (aromatase), myostatin, and prolyl hydroxylase-2 (PHD2).

Parameter	Protodioscin			Diosgenin			DMF			TMF		
	CYP19A1	Myostatin	PHD	CYP19A1	Myostatin	PHD	CYP19A1	Myostatin	PHD	CYP19A1	Myostatin	PHD
Van der waals	15	14	5	7	7	5	6	10	9	4	4	5
Conventional hydrogen bonds	6	5	2				1		1	2		2
Carbon hydrogen bonds	2	1				1		1			1	
Alkyl	3		1	2	4	3	1	2	1	8	2	1
π -alkyl	2	2	1	2				2	3	6	3	1
π -cation			2				3	1				2
π -donor								1				
Amide π -stacked										1		
π -sigma									1			
π -sulfur									1	1		
π - π stacked							1		2		2	
π - π T shaped							1				2	2
π - Anion												

Diosgenin displayed a far more hydrophobic interaction signature, with its binding profile dominated by alkyl contacts and van der Waals forces. In CYP19A1, these interactions included seven van der Waals contacts and four alkyl contacts, while similar patterns emerged in myostatin (seven van der Waals and two alkyl contacts) and PHD2 (five van der Waals and three alkyl contacts). The scarcity of hydrogen bonds or polar interactions across all three targets reflects the rigid hydrophobic steroidal structure of diosgenin, which is known to insert deeply into lipid-rich or hydrophobic protein pockets. This hydrophobic anchoring mechanism is consistent with well-documented ability of the diosgenin to influence hormone biosynthesis, androgen–estrogen balance, and anabolic signaling pathways^47,73,104,107,123,124^. Its consistent insertion into hydrophobic sites across all three proteins suggests that diosgenin may enhance the effects of the Testolift by stabilizing protein conformations and modulating catalytic surfaces through sterically driven hydrophobic reinforcement rather than polar interactions.

The interaction patterns observed for DMF introduce a greater degree of aromatic complexity. While DMF formed a modest number of van der Waals contacts across CYP19A1, myostatin, and PHD2 (five, six, and ten, respectively), its defining feature was the presence of prominent π-based interactions. These included π–alkyl interactions, π–cation bonding, π–donor hydrogen bonding, and π–π stacking, particularly strong in PHD2 where DMF engaged aromatic and sulfur-containing residues through π–π stacking and π–sulfur interactions. The presence of π–cation interactions in myostatin and PHD2 suggests that DMF is attuned to electrostatically rich microenvironments, allowing it to modulate regulatory proteins that depend on aromatic or charged residues for functionality. This interaction pattern is consistent with previous reports demonstrating that flavones and polymethoxyflavones modulate myostatin and PHD-family enzymes through aromatic and redox-mediated interactions^39,110,111,114,120,121,125–127^. Given this profile, DMF likely contributes to the physiological effects of the Testolift by stabilizing or interfering with aromatic residue clusters essential for myostatin binding and PHD2 catalytic turnover, ultimately promoting enhanced muscle function and oxygen-efficient metabolism.

TMF, with its additional methoxy substitution, demonstrated the most structurally diverse interaction profile of all four compounds. In CYP19A1, TMF formed multiple van der Waals contacts together with alkyl, π-alkyl, and amide–π stacking interactions. In myostatin, the ligand displayed dual π–π T-shaped interactions with Tyr80 and additional π–π stacking with Trp25, highlighting the role of its planar aromatic system in engaging aromatic pockets involved in protein–protein recognition. The PHD2 interactions were even more elaborate, with TMF forming hydrogen bonds, π–alkyl contacts, π–π interactions, π-anion contacts with Asp315, and π-cation interactions with His313. This combination of charged and aromatic interactions along with hydrophobic stabilization suggests a unique capacity to interfere precisely with the catalytic machinery of PHD2, which relies on aromatic and basic residues near the HIF-1α recognition interface. Such behavior is consistent with evidence showing that polymethoxyflavones inhibit prolyl hydroxylases by stabilizing aromatic residues and altering redox conditions within the active site^38,39,98–101,108,111,119,121,125^. Collectively, TMF appears to serve as a highly specialized regulatory ligand capable of fine-tuning enzyme activity through multi-mode aromatic and electrostatic engagement.

Comparing these four bioactive compounds reveals a cohesive but differentiated functional architecture within the Testolift formulation. Protodioscin emerges as the broad-spectrum binder, engaging all three targets through extensive polar and nonpolar interactions that enable it to anchor firmly within diverse catalytic and allosteric sites. This widespread interaction capability supports its role in modulating testosterone availability, suppressing myostatin, and stabilizing hypoxia-response pathways. Diosgenin, in contrast, consistently employs hydrophobic insertion as its principal mechanism, reinforcing structural regions of enzymes involved in steroidogenesis and muscle regulation through sterol-like anchoring that is distinct from the polar-driven behavior of protodioscin. DMF contributes a more nuanced aromatic mode of action, supporting regulatory interference at aromatic and electrostatic hot spots within myostatin and PHD2, thereby enhancing muscular contractility and oxygen utilization. TMF, with the richest array of π-mediated interactions, demonstrates the highest degree of catalytic precision by combining hydrogen bonding with aromatic, anionic, and cationic interactions across CYP19A1, myostatin, and PHD2, reflecting its role as a potent and selective modulator within the formulation.

Together, these compounds create a multilayered network of complementary binding mechanisms that support the tri-target formulation design of Testolift. The synergy arises not merely from additive effects but from coordinated molecular specialization, where each compound targets distinct physicochemical microenvironments within key proteins governing testosterone balance, muscle anabolism, and oxygen-dependent metabolic efficiency.

### Binding affinity evaluation of Testolift phytochemicals across CYP19A1, myostatin, and PHD2

The binding affinity analysis of the four Testolift phytochemicals revealed clear structural trends governing ligand–protein interactions across the three mechanistically important targets such as CYP19A1, myostatin, and PHD2 (Table 7). Protodioscin demonstrated the strongest affinity for CYP19A1 with a binding energy of − 9.2 kcal/mol, surpassing diosgenin (− 7.7 kcal/mol), DMF (− 7.9 kcal/mol), and TMF (− 8.1 kcal/mol). This pronounced affinity is consistent with the extensive hydrogen bonding, π–alkyl contacts, and van der Waals stabilization observed in the interaction mapping of protodioscin, suggesting a robust inhibitory engagement with the aromatase catalytic cavity. These characteristics align with prior reports indicating that steroidal saponins engage aromatase and related steroidogenic enzymes through multivalent surface interactions and polar anchoring mechanisms^50,58,95,96,104,123,125^.

**Table 7:** Molecular docking–derived binding affinities (kcal/mol) of protodioscin, diosgenin, 5,7-dimethoxyflavone (DMF), and 5,7,4′-trimethoxyflavone (TMF) against CYP19A1, myostatin, and prolyl hydroxylase-2 (PHD2).

Components	CYP19A1	Myostatin	PHD
Protodisocin	− 9.2	− 7.1	− 7.6
Diosgenin	− 7.7	− 7.7	− 7.9
DMF	− 7.9	− 6.9	− 7.5
TMF	− 8.1	− 6.2	− 6.7

In the case of myostatin, diosgenin emerged as the strongest binder (− 7.7 kcal/mol), slightly outperforming protodioscin (− 7.1 kcal/mol), DMF (− 6.9 kcal/mol), and TMF (− 6.2 kcal/mol). The superior affinity of diosgenin corresponds to its deep hydrophobic insertion into the myostatin inhibitory groove, supported by multiple alkyl and van der Waals contacts. The affinity profile is consistent with literature describing how hydrophobic steroidal ligands preferentially bind to regulatory surfaces of growth differentiation factor family proteins such as myostatin, stabilizing inactive conformations that suppress catabolic signaling^62,102,104,126,128,129^. The moderate affinities of DMF and TMF reflect their aromatic stacking mechanisms which, while effective, do not substitute for deep hydrophobic insertion within the relatively nonpolar myostatin binding cleft.

For PHD2, diosgenin again exhibited the strongest binding affinity (− 7.9 kcal/mol), followed closely by protodioscin (− 7.6 kcal/mol), DMF (− 7.5 kcal/mol), and TMF (− 6.7 kcal/mol). PHD2 contains a heterogeneous catalytic domain composed of both hydrophobic and aromatic residues surrounding the Fe²⁺-dependent active site. The strong binding of diosgenin and protodioscin reflects their capacity to engage hydrophobic pockets adjacent to HIF-1α recognition residues, whereas the moderate affinities of DMF and TMF arise from their aromatic and π-mediated interactions with catalytic and neighboring residues. These findings are consistent with the known susceptibility of PHD-family enzymes to both hydrophobic and π-electron–rich dietary phytochemicals that modulate HIF stability under normoxic conditions^39,47,114,124,127,130–132^.

Overall, the binding affinity landscape indicates a complementary pattern among the Testolift phytochemicals. Protodioscin dominates CYP19A1 binding, supporting its key role in testosterone preservation through aromatase modulation. Diosgenin displays the highest affinity for both myostatin and PHD2, suggesting a dual role in suppressing muscle atrophy signaling and enhancing oxygen-responsive metabolic pathways. DMF and TMF exhibit moderate but structurally rich affinities across all targets, functioning as aromatic modulators that reinforce the activity of the steroidal ligands by stabilizing aromatic and electrostatically active pockets. These combined behaviors validate the molecular logic behind the tri-target formulation strategy of Testolift, wherein ligand diversity ensures broad biochemical coverage and synergistic enhancement of endocrine, muscular, and hypoxia-adaptive physiology.

### Molecular dynamics results for the CYP19A1–ligand complexes

**Fig. 14:**
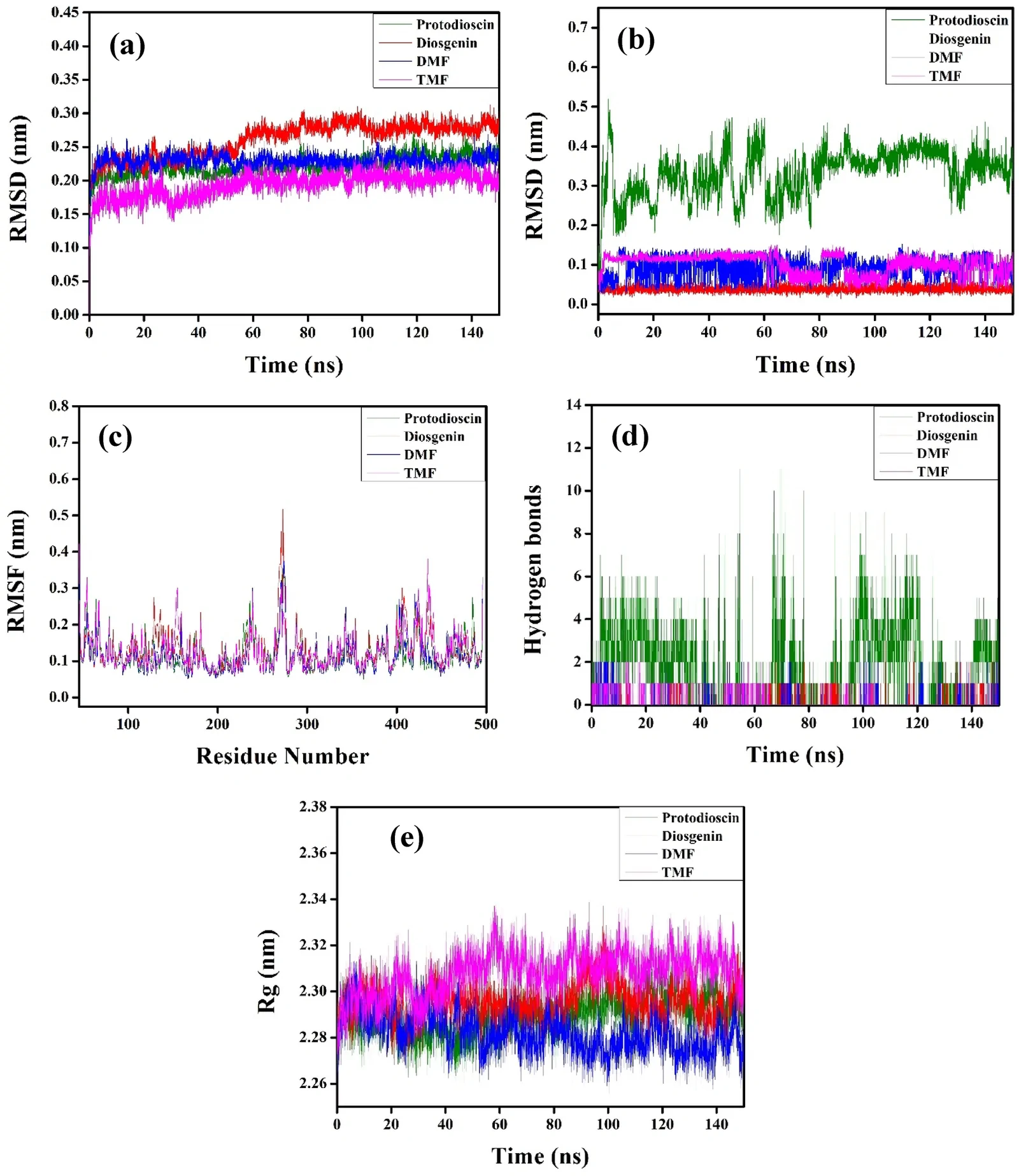
Molecular dynamics simulation analysis of CYP19A1 in complex with the Testolift phytochemicals protodioscin (green), diosgenin (red), 5,7-dimethoxyflavone (DMF, blue), and 5,7,4′-trimethoxyflavone (TMF, pink) over a 150 ns trajectory. (**a**) Root mean square deviation (RMSD) of the CYP19A1 backbone, illustrating ligand-dependent effects on global structural stability. (**b**) Ligand RMSD showing internal conformational fluctuation of each phytochemical within the active-site cavity. (**c**) Root mean square fluctuation (RMSF) of CYP19A1 residues highlighting localized flexibility changes in response to ligand binding. (**d**) Temporal hydrogen-bonding profiles depicting the persistence and frequency of polar interactions between each ligand and CYP19A1. (**e**) Radius of gyration (Rg) demonstrating ligand-specific influences on overall protein compactness throughout the simulation.

The molecular dynamics (MD) simulations provided detailed insight into the conformational stability and interaction persistence of CYP19A1 in complex with the four Testolift bioactive constituents (Fig. 14). The protein RMSD profiles demonstrated that CYP19A1 remained structurally stable throughout the 150 ns simulation for all systems, though the degree of fluctuation varied depending on the ligand (Fig. 14a). The diosgenin complex (red) maintained the lowest RMSD values, remaining close to 0.20 nm for almost the entire trajectory, indicative of minimal conformational perturbation and strong conformational compatibility with the active-site cavity. In contrast, the protodioscin complex (green) demonstrated the highest RMSD excursions approaching 0.35–0.40 nm reflecting ligand-induced local rearrangements within the active site caused by its bulky and flexible glycosidic architecture. The DMF (blue) and TMF (pink) complexes exhibited intermediate RMSD stability, maintaining values between 0.22 and 0.28 nm, suggesting moderate structural adjustments consistent with the aromatic ligands occupying a stable but adaptable binding conformation. These findings are consistent with prior observations that cytochrome P450 enzymes accommodate both rigid lipophilic ligands and flexible polar ligands through distinct structural rearrangement patterns^133^.

The ligand RMSD profiles revealed a parallel trend in ligand stability (Fig. 14b). Protodioscin (green) displayed the highest ligand RMSD and the greatest internal fluctuations, confirming extensive conformational mobility inside the binding pocket. This behavior is expected given the high flexibility of its sugar moieties and the large sterol–glycoside architecture, which must continuously reposition to maintain optimal contacts. Diosgenin (red), in contrast, exhibited the lowest ligand RMSD, maintaining exceptional stability due to its rigid steroidal skeleton and deep hydrophobic embedding in the catalytic cleft. The polymethoxyflavones showed moderate fluctuations: DMF (blue) maintained a relatively low RMSD profile with high positional stability, whereas TMF (pink) exhibited slightly greater fluctuations than DMF, likely due to the additional methoxy substitution conferring enhanced rotational freedom. These observations are consistent with the stability trend reported for flavones binding within P450-type hydrophobic and aromatic pockets^98–101^.

Residue-level flexibility of CYP19A1 was assessed using RMSF analysis to evaluate the structural response to ligand binding (Fig. 14c). Across all complexes, RMSF values remained below 0.15 nm for the majority of residues, indicating minimal perturbation of local dynamics. Elevated fluctuations were primarily confined to solvent-exposed loop regions, most notably within residues ~ 250–280, which correspond to flexible surface loops distal to the conserved catalytic α-helical core. In contrast, residues forming the central P450 fold including the heme-binding region and surrounding α-helices spanning approximately residues 300–470 exhibited consistently low RMSF values across all systems. No abnormal spikes or signatures of localized unfolding were observed in any ligand-bound complex, demonstrating that protodioscin, diosgenin, DMF, and TMF do not induce destabilizing conformational changes. These results underscore the intrinsic structural robustness of CYP19A1 and are consistent with the well-established tolerance of cytochrome P450 enzymes to chemically diverse ligands^133,134^.

Hydrogen bond dynamics displayed marked differences among the four ligands (Fig. 14d). Protodioscin (green) consistently formed the highest number of hydrogen bonds often exceeding 6–12 persistent interactions during the simulation due to its extensive network of hydroxyl and glycosidic functionalities. These stable polar contacts anchor protodioscin deeply within the active site. Diosgenin (red) formed only sporadic one-to-two hydrogen bonds, consistent with its largely hydrophobic interaction mode. DMF (blue) and TMF (pink) maintained intermittent hydrogen bonds, generally between 1 and 3, reflecting the dominance of π-mediated interactions over classical hydrogen bonding in their binding mechanism. This behavior aligns with substituted flavones known for π–π stacking and hydrophobic modulation rather than strong hydrogen-bond-driven binding^101^.

Hydrogen-bonding analysis revealed clear ligand-dependent interaction patterns within the active site. Protodioscin exhibited the highest average number of hydrogen bonds across the simulation, driven by its multiple hydroxyl and glycosidic groups, resulting in frequent but highly dynamic polar interactions with residues lining the binding cavity. These hydrogen bonds formed and dissociated continuously, indicating flexible anchoring rather than rigid fixation, and suggesting a potential entropic cost associated with maintaining multiple transient contacts. In contrast, diosgenin formed only sporadic hydrogen bonds, typically limited to one or two short-lived interactions, consistent with a predominantly hydrophobic binding mode. The flavone derivatives DMF and TMF displayed intermediate behavior, maintaining low numbers of intermittent hydrogen bonds (generally one to three) while relying primarily on π-mediated and hydrophobic interactions for stabilization. Collectively, these results indicate that although all ligands preserve the structural integrity of CYP19A1, they differ markedly in hydrogen-bond dynamics and in the balance between polar interactions and conformational flexibility within the active site.

The radius of gyration (Rg) profiles further confirmed ligand-dependent alterations in global protein compactness (Fig. 14e). The TMF complex (pink) exhibited the highest Rg values across the simulation, indicating a slightly expanded protein conformation and modestly increased flexibility. Diosgenin (red) also displayed higher-than-average Rg values, consistent with the structural adjustments observed in its RMSD trajectory. In contrast, protodioscin (green) maintained a consistently low and stable Rg profile, demonstrating that its extensive hydrogen-bond network promotes a compact CYP19A1 structure. DMF (blue) showed one of the most compact and stable Rg profiles, reflecting tight structural packing resulting from its strong conformational compatibility with the active-site topology. Together, these trends indicate that TMF and diosgenin induce modest enhancements in global flexibility, while protodioscin and DMF stabilize more compact protein conformations.

Collectively, the assessed MD analysis demonstrates that the four phytochemicals stabilize the CYP19A1 structure through distinct interaction modes. Protodioscin stabilizes CYP19A1 via extensive hydrogen bonding but induces local flexibility due to its large, flexible scaffold. Diosgenin binds rigidly and deeply within the hydrophobic pocket, minimizing ligand mobility and maintaining global structural stability. DMF achieves the most structurally harmonious binding, producing minimal protein or ligand fluctuation. TMF, while stable, encourages slightly higher global flexibility due to its expanded aromatic substitution pattern. These mechanistic differences offer a complementary molecular rationale for the inclusion of multiple structurally diverse phytochemicals in the Testolift formulation, supporting synergistic modulation of aromatase through orthogonal stabilizing mechanisms.

### Molecular dynamics analysis of myostatin–ligand complexes

**Fig. 15:**
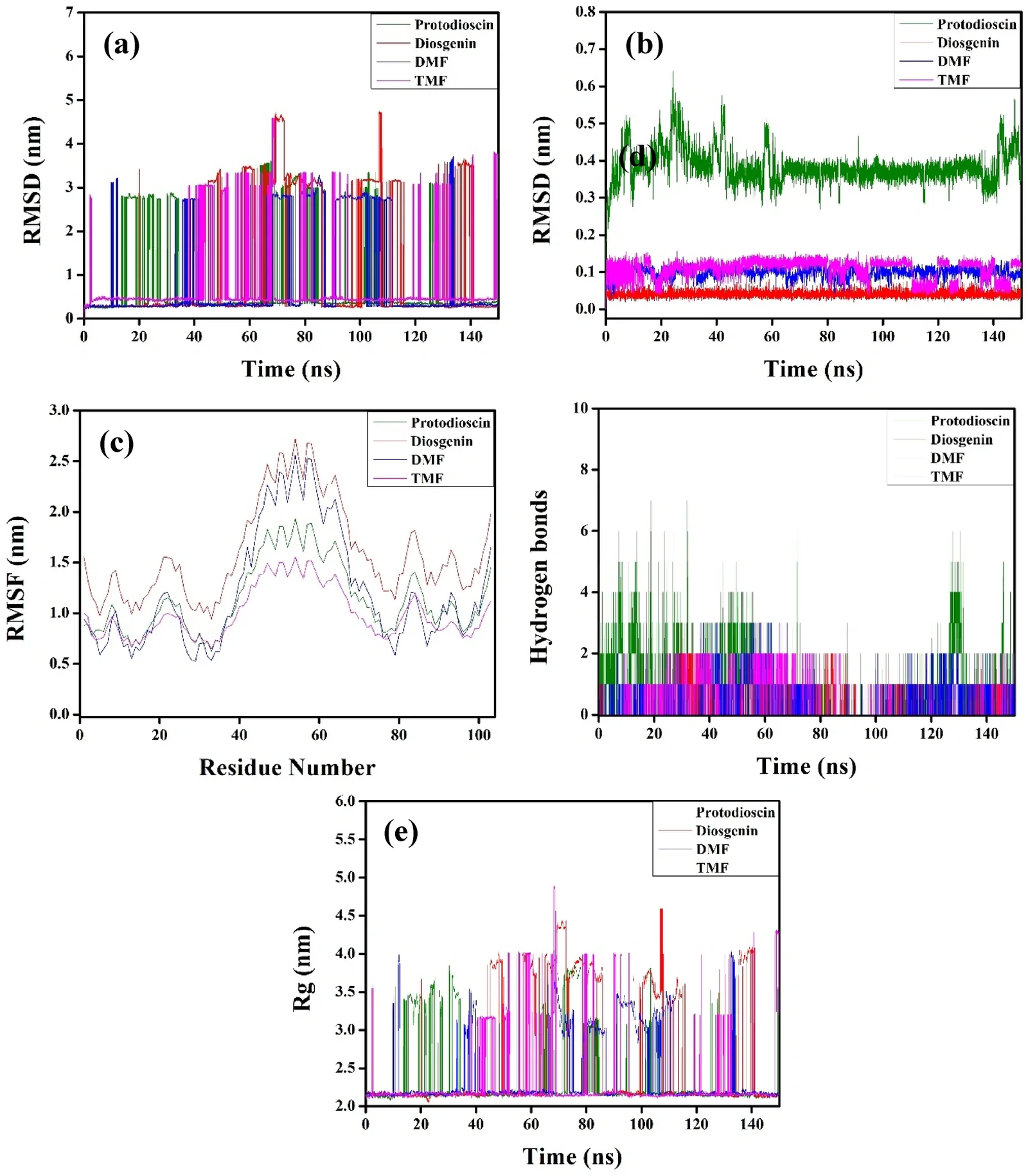
Molecular dynamics simulation analysis of Myostatin (GDF-8) in complex with Testolift phytochemicals protodioscin (green), diosgenin (red), 5,7-dimethoxyflavone (DMF, blue), and 5,7,4′-trimethoxyflavone (TMF, pink) over a 150 ns simulation period. (**a**) Root mean square deviation (RMSD) of the Myostatin backbone illustrating ligand-dependent effects on global protein stability. (**b**) Ligand RMSD depicting the conformational stability and internal flexibility of each bioactive compound within the Myostatin binding region. (**c**) Root mean square fluctuation (RMSF) of individual Myostatin residues highlighting localized flexibility changes induced by ligand binding. (**d**) Time-dependent hydrogen-bond analysis showing the persistence and frequency of polar interactions between Myostatin and each ligand. (**e**) Radius of gyration (Rg) profiles demonstrating ligand-specific influences on overall protein compactness and conformational dynamics throughout the simulation.

To further elucidate the dynamic stability and mechanistic behavior of Testolift phytochemicals in modulating Myostatin, molecular dynamics simulations were conducted for 150 ns on Myostatin complexes with protodioscin, diosgenin, DMF, and TMF (Fig. 15). The protein RMSD analysis revealed distinct ligand-dependent conformational responses of Myostatin (Fig. 15a). The diosgenin-bound complex exhibited the highest RMSD excursions and frequent transient spikes, indicating greater backbone rearrangements and reduced global stability. This behavior likely arises from the rigid, hydrophobic steroidal scaffold of diosgenin, which inserts deeply into nonpolar regions of the protein and necessitates adaptive conformational adjustments of the surrounding residues. In contrast, DMF- and TMF-bound complexes maintained comparatively lower and more stable RMSD values throughout the simulation, suggesting improved structural compatibility with the Myostatin binding surface. The protodioscin complex displayed intermediate RMSD behavior, reflecting a balance between stabilizing interactions and conformational flexibility imposed by its bulky saponin structure^38,111,123^.

Ligand RMSD profiles further differentiated the stability of the phytochemicals within the Myostatin binding region (Fig. 15b). Protodioscin showed the highest ligand RMSD, indicating significant internal flexibility and continuous conformational adjustment of its glycosidic chains during the simulation. Diosgenin, by contrast, exhibited the lowest ligand RMSD values, reflecting exceptional positional stability driven by its compact and rigid steroidal core. DMF and TMF displayed moderate ligand RMSD values, with TMF showing slightly higher fluctuations than DMF. This difference can be attributed to the additional methoxy group in TMF, which increases rotational freedom and dynamic responsiveness while maintaining stable binding. These findings align with previous reports demonstrating that rigid sapogenins exhibit reduced ligand mobility, whereas polymethoxyflavones display controlled flexibility within protein binding pockets^50,102,111,123^.

Residue-level flexibility was assessed using RMSF analysis, which demonstrated that ligand binding did not induce local or global destabilization of Myostatin (Fig. 15c). Across all complexes, residue fluctuations were predominantly localized to surface-exposed loop regions most notably residues ~45–65 corresponding to an exposed loop between β-strands in the growth factor domain and to flexible N- and C-terminal segments, while the disulfide-stabilized cystine-knot core remained structurally rigid. The Diosgenin-bound complex exhibited the highest RMSF values within the central loop region, indicating enhanced local flexibility likely associated with weaker anchoring or shallow binding. In contrast, DMF- and TMF-bound complexes showed consistently lower RMSF amplitudes in the same regions, reflecting more effective restraint of local motions. Protodioscin displayed an intermediate RMSF profile, characterized by localized flexibility that did not propagate into the protein core, consistent with its larger molecular size and conformational mobility. Overall, RMSF trends support differential ligand-induced modulation of loop flexibility without compromising the structural integrity of the Myostatin core.

Hydrogen-bond analysis highlighted marked differences in interaction persistence among the ligands (Fig. 15d). Protodioscin consistently formed the highest number of hydrogen bonds throughout the simulation, often maintaining multiple simultaneous interactions, reflecting its abundance of hydroxyl groups and glycosidic functionalities. These persistent polar contacts contribute significantly to anchoring protodioscin within the Myostatin binding region. Diosgenin formed very few hydrogen bonds, confirming that its interaction with Myostatin is predominantly hydrophobic. DMF and TMF displayed intermittent hydrogen bonding, typically maintaining one to two interactions over time, indicating that their binding stability relies primarily on hydrophobic and π-mediated interactions rather than extensive polar contacts^38,50,102,104,110,111,123^.

The radius of gyration (Rg) profiles indicated that Myostatin retained a folded core throughout the 150 ns simulations but exhibited pronounced ligand-dependent global fluctuations (Fig. 15e). All complexes showed recurrent Rg expansions, reflecting substantial conformational breathing rather than a rigidly compact state. TMF- and diosgenin-bound systems displayed higher and more frequent Rg excursions, consistent with enhanced global flexibility. In contrast, protodioscin and DMF showed comparatively lower average Rg values, indicating a tendency toward more compact conformational ensembles, albeit without achieving a fully stabilized equilibrium. Overall, the Rg analysis suggests that ligand binding modulates global flexibility of Myostatin rather than enforcing strict structural stabilization.

Collectively, the molecular dynamics results demonstrate that the Testolift phytochemicals modulate Myostatin through complementary dynamic mechanisms. Diosgenin provides highly stable ligand positioning but induces greater protein rearrangement; protodioscin offers strong polar stabilization coupled with conformational flexibility; and DMF and TMF achieve balanced stabilization through aromatic and hydrophobic interactions while minimizing structural perturbation. This synergistic dynamic behavior supports the rationale for combining these bioactives in a multi-component formulation aimed at suppressing Myostatin-mediated muscle catabolism and enhancing muscle performance without compromising protein structural integrity.

### Molecular dynamics evaluation of prolyl hydroxylase-2–ligand complexes and oxygen-sensing modulation

**Fig. 16:**
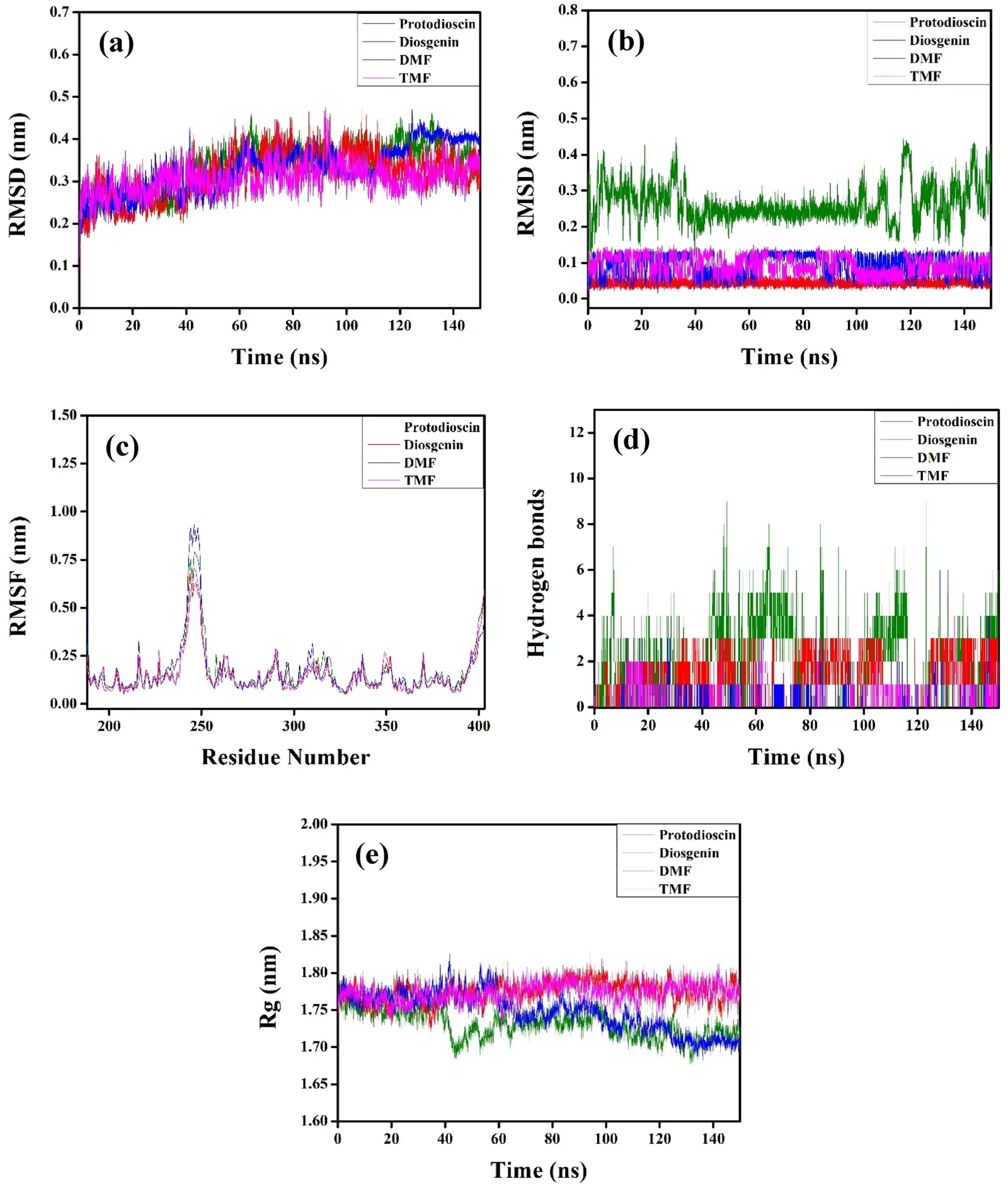
Molecular dynamics simulation analysis of prolyl hydroxylase-2 (PHD2) in complex with Testolift bioactive compounds protodioscin (green), diosgenin (red), 5,7-dimethoxyflavone (DMF, blue), and 5,7,4′-trimethoxyflavone (TMF, pink) over a 150 ns simulation period. (**a**) Root mean square deviation (RMSD) of the PHD2 backbone illustrating ligand-dependent effects on global protein stability. (**b**) Ligand RMSD depicting the conformational stability and internal flexibility of each bioactive compound within the PHD2 binding pocket. (**c**) Root mean square fluctuation (RMSF) of PHD2 residues highlighting localized flexibility changes induced by ligand binding. (**d**) Time-dependent hydrogen-bond analysis showing the frequency and persistence of polar interactions between PHD2 and each ligand. (**e**) Radius of gyration (Rg) profiles demonstrating ligand-specific influences on overall protein compactness and conformational dynamics throughout the simulation.

Molecular dynamics simulations were carried out to investigate the conformational stability and interaction dynamics of prolyl hydroxylase-2 (PHD2) in complex with protodioscin, diosgenin, DMF, and TMF, providing mechanistic insight into the potential modulation of the HIF-1α oxygen-sensing pathway (Fig. 16). Analysis of the protein backbone RMSD revealed that all complexes reached equilibrium early in the simulation and remained structurally stable throughout the 150 ns trajectory (Fig. 16a). However, ligand-dependent differences were evident. The protodioscin-bound complex (green) exhibited slightly higher RMSD values toward the latter stages of the simulation, indicating moderate conformational adaptation of PHD2 in response to the bulky and flexible saponin scaffold. In contrast, diosgenin (red) and DMF (blue) maintained comparatively lower RMSD values, suggesting enhanced backbone stability, while TMF (pink) displayed intermediate RMSD behavior consistent with its aromatic but moderately flexible structure^39,114,117,124,127,135^.

Ligand RMSD analysis highlighted pronounced differences in ligand mobility within the PHD2 binding pocket (Fig. 16b). Protodioscin showed the highest ligand RMSD, reflecting substantial internal flexibility and continuous repositioning of its glycosidic moieties during the simulation. Diosgenin, in contrast, exhibited the lowest ligand RMSD, indicating strong positional stability driven by its rigid steroidal backbone and favorable hydrophobic complementarity with the PHD2 active-site environment. DMF and TMF demonstrated moderate ligand RMSD values, with TMF displaying slightly greater fluctuations than DMF, likely due to the additional methoxy substitution increasing rotational freedom. These trends are consistent with reported dynamics of small-molecule modulators interacting with Fe²⁺-dependent dioxygenases such as PHD2^39,114,117,124,127,135,136^.

Residue-level RMSF analysis indicated that ligand binding did not compromise the local structural stability of PHD2 (Fig. 16c). Across all complexes, most residues exhibited RMSF values below 0.2 nm, confirming preservation of a stable protein core. Fluctuations were primarily restricted to surface-exposed loop regions, with a prominent peak observed around residues ~ 240–255, corresponding to an intrinsically flexible loop. The diosgenin-bound complex showed the highest RMSF peak (~ 0.8–0.9 nm) in this region, indicating weaker local restraint. DMF- and TMF-bound complexes displayed reduced fluctuations, reflecting enhanced local stabilization of the loop. The protodioscin-bound complex exhibited intermediate RMSF behavior, consistent with localized flexibility required to accommodate a bulky ligand without propagation of instability. Increased fluctuations at the terminal residues (~ 380–400) were observed in all systems and are attributable to solvent exposure rather than ligand-induced effects. These observations align with the intrinsic structural robustness of PHD2 and its ability to tolerate diverse ligand chemotypes without loss of fold integrity^12,39,114,117,119,124,127^.

Hydrogen-bond analysis revealed clear distinctions among the ligands (Fig. 16d). Protodioscin consistently formed the highest number of hydrogen bonds with PHD2 throughout the simulation, reflecting its multiple hydroxyl and sugar functionalities and contributing to strong polar anchoring within the active site. Diosgenin formed fewer hydrogen bonds, indicating a predominantly hydrophobic mode of interaction. DMF and TMF displayed intermittent hydrogen bonding, generally maintaining one to three interactions over time, consistent with their reliance on π-mediated and hydrophobic contacts rather than extensive polar networks. Such differences in hydrogen-bonding behavior are characteristic of steroidal saponins, sapogenins, and polymethoxyflavones interacting with oxygen-sensing enzymes^39,114,117,119,124,127,137^.

The radius of gyration analysis further supported ligand-dependent effects on global protein compactness (Fig. 16e). Protodioscin (green) showed a slight decrease in Rg values over time, indicating a tendency toward a more compact PHD2 conformation stabilized by extensive hydrogen-bonding interactions. DMF (blue) similarly maintained low and stable Rg values, reflecting tight structural packing. In contrast, diosgenin (red) and TMF (pink) exhibited slightly higher Rg values, suggesting modest increases in global flexibility or transient expansion of the protein structure. Importantly, none of the ligands induced destabilizing changes, underscoring the overall structural resilience of PHD2 during ligand engagement.

Collectively, the molecular dynamics results demonstrate that the Testolift phytochemicals engage PHD2 through distinct yet coordinated dynamic interaction mechanisms. Protodioscin provides extensive polar stabilization accompanied by increased ligand flexibility, diosgenin exhibits comparatively rigid and stable hydrophobic binding with minimal internal motion, while DMF and TMF achieve balanced stabilization through aromatic and hydrophobic interactions. These ligand-specific dynamic behaviors support the possibility that combined modulation of PHD2-associated signaling pathways may contribute to HIF-1α stabilization, cellular oxygen utilization, and metabolic adaptation relevant to endurance physiology and mitochondrial function.

Taken together, these findings support a multi-ligand interaction framework in which structurally diverse phytochemicals collectively engage multiple peripheral pathways associated with testosterone regulation, muscle physiology, and oxygen-dependent metabolic adaptation. Rather than functioning through direct hormonal replacement or central endocrine manipulation, the Testolift formulation appears to operate through coordinated modulation of testosterone-to-estradiol conversion, myostatin-associated catabolic signaling, and PHD2-regulated oxygen-sensing pathways. The distinct physicochemical characteristics, pharmacokinetic profiles, and target-specific interaction behaviors observed among protodioscin, diosgenin, DMF, and TMF further support the possibility of complementary and potentially synergistic pathway modulation within the Testolift formulation. Although the docking affinities observed in this study fall within a relatively narrow range and do not establish definitive target selectivity, the distinct interaction architectures and dynamic behaviors observed across the three protein systems provide a mechanistic rationale for potential complementary biological activity. Nevertheless, the present findings remain computational in nature, and further experimental validation through in vitro, in vivo, and clinical investigations will be necessary to confirm the proposed biological mechanisms, target modulation, and physiological relevance associated with testosterone regulation, muscle anabolic signaling, and endurance-related metabolic adaptation.

## Conclusion

The present in silico investigation provides a comprehensive molecular and pharmacokinetic rationale supporting the tri-target design of the Testolift nutraceutical formulation for testosterone preservation, muscle anabolism, and endurance-related physiological enhancement. By integrating physicochemical profiling, ADMET prediction, molecular docking, and molecular dynamics simulations, this study demonstrates that the selected phytochemicals such as protodioscin, diosgenin, 5,7-dimethoxyflavone (DMF), and 5,7,4′-trimethoxyflavone (TMF) exhibit complementary interaction profiles with aromatase (CYP19A1), myostatin, and prolyl hydroxylase-2 (PHD2), collectively addressing key peripheral mechanisms underlying testosterone decline and impaired physical performance.

Protodioscin emerged as a broad-spectrum surface-active modulator characterized by extensive hydrogen bonding and van der Waals interactions across all three targets, particularly within the aromatase catalytic environment. Its highly polar and high-molecular-weight profile together with favorable safety predictions support a mechanism centered on extracellular or membrane-associated modulation of steroidogenic enzymes rather than direct endocrine disruption. Diosgenin demonstrated strong hydrophobic insertion and favorable interaction characteristics within myostatin and PHD2, alongside meaningful androgen receptor interaction probabilities consistent with steroidogenic and anabolic signaling. The polymethoxyflavones DMF and TMF exhibited the most favorable drug-like physicochemical and absorption characteristics, efficient membrane permeability, and π-driven interaction profiles supporting modulation of myostatin and PHD2-associated pathways related to mitochondrial function, antioxidant signaling, and endurance physiology.

The ADMET and toxicity assessments further indicated a favorable overall safety landscape, including low predicted mutagenicity, carcinogenicity, and clinical toxicity across all compounds. Molecular dynamics simulations confirmed stable ligand–protein complexes with sustained binding behavior, limited structural perturbation, and favorable conformational stability across CYP19A1, myostatin, and PHD2 systems.

Taken together, these findings establish a mechanistically coordinated multi-target interaction framework in which each bioactive compound fulfills a distinct yet potentially synergistic role. Rather than relying on direct hormonal replacement or central endocrine manipulation, the Testolift formulation appears to operate through coordinated peripheral modulation of testosterone-to-estradiol conversion, suppression of myostatin-mediated muscle catabolism, and regulation of oxygen-sensing and mitochondrial pathways. Compared with conventional single-target testosterone-supportive nutraceutical approaches, the Testolift framework was designed to integrate complementary modulation of testosterone preservation, muscle anabolic signaling, and endurance-related metabolic adaptation through structurally distinct phytochemical interactions.

Although the present findings provide important mechanistic insight into the coordinated multi-target interaction potential of Testolift phytochemicals, the study remains computational in nature. Therefore, further experimental validation through in vitro, in vivo, and clinical investigations will be necessary to confirm the proposed biological mechanisms, target modulation, and translational relevance associated with testosterone regulation, muscle anabolic signaling, and endurance-related metabolic adaptation. Nevertheless, the current in silico evidence strongly supports the mechanistic plausibility and nutraceutical potential of the Testolift tri-target framework for testosterone and performance support.

## Data Availability

All data supporting the findings of this study are available within the article.
